# Neuroinflammation and acute ischemic stroke: impact on translational research and clinical care

**DOI:** 10.3389/fsurg.2025.1501359

**Published:** 2025-04-28

**Authors:** Simon Levinson, Benjamin Pulli, Jeremy J. Heit

**Affiliations:** ^1^ Department of Neurosurgery, School of Medicine, Stanford University, Stanford, CA, United States; ^2^ Department of Radiology, School of Medicine, Stanford University, Stanford, CA, United States

**Keywords:** stroke, immune system, stem cells, neuroinflammation, cerebral ischemia

## Abstract

**Background:**

Stroke, encompassing both ischemic and hemorrhagic subtypes, is a leading cause of mortality and disability globally and current treatments remain limited. Neuroinflammation plays a crucial role in the pathophysiology of stroke, influencing both acute injury and long-term recovery.

**Objective:**

This review aims to provide a comprehensive overview of neuroinflammation in stroke, detailing the mechanisms, clinical implications, and potential therapeutic strategies.

**Methods:**

A detailed literature review was conducted, focusing on recent advancements in understanding the neuroinflammatory processes in stroke, including the roles of thromboinflammation, blood-brain barrier (BBB) disruption, and the immune response.

**Results:**

The initial ischemic insult triggers an inflammatory cascade involving both innate and adaptive immune responses. BBB disruption allows peripheral immune cells and neurotoxic substances to infiltrate the brain, exacerbating neuronal damage and increasing the risk of infections such as pneumonia and urinary tract infections. Thromboinflammation, characterized by platelet activation and immune cell interactions, further complicates the ischemic environment. Proteomic studies have identified key biomarkers that offer insights into neuroinflammatory mechanisms and potential therapeutic targets. Advances in imaging techniques, such as PET and MRI, enable real-time monitoring of neuroinflammation, facilitating personalized treatment approaches.

**Conclusion:**

Neuroinflammation significantly impacts stroke outcomes, presenting both challenges and opportunities for treatment. Current immunologic therapeutic strategies are limited. Future research should aim to further elucidate the complex immune interactions in stroke, refine imaging biomarkers for clinical use, and develop effective interventions to mitigate neuroinflammation.

## Introduction

Stroke is a major cause of mortality and disability within the United States, presenting as either ischemic or hemorrhagic subtypes. Ischemic strokes constitute approximately 85% of cases and primarily result from occlusions in cerebral blood flow, while the remaining 15% are hemorrhagic due to arterial ruptures (1–3). Both types of strokes pose significant public health challenges, contributing to substantial morbidity and mortality nationwide. Annually, approximately 795,000 individuals in the United States experience a stroke, with 610,000 being first-time events (1, 4, 5). The mortality rate is approximately 25% for ischemic strokes and nearly 50% for hemorrhagic strokes (1, 2). The incidence of ischemic stroke correlates positively with advancing age, though it poses a risk at all stages of life (2, 3, 6). Economically, stroke imposes a substantial burden, with combined direct and indirect costs surpassing $46 billion annually in the United States. These costs encompass healthcare services, pharmacological treatments, and loss of productivity (7, 8).

Significant advancements have been made in the acute management of stroke in recent years. These include expanding the therapeutic window for intravenous tissue plasminogen activator (tPA) or Tenecteplase (TNK), developing new imaging techniques such as perfusion imaging to better identify salvageable brain tissue, and the widespread use of endovascular mechanical thrombectomy (EVT) up to 24 h from stroke onset (9–12). Despite the effectiveness of these treatments, a large subset of patients fail to benefit from these therapies or do not access care within the therapeutic window for intravenous thrombolysis or EVT (13). Therefore, there is an urgent need to investigate novel treatments for stroke.

In ischemic stroke, an obstruction in a cerebral vessel leads to oxygen deprivation of the brain tissue supplied by the thrombosed vessel, resulting in a multi-phased death of cells of the compromised neurovascular unit. Several mechanisms of cell death occur, including free radical damage, oxidative stress, neuronal excitotoxicity, mitochondrial senescence, and a disordered acute and chronic inflammatory response. While the primary insult from the initial ischemic event causes a certain amount of irreversible damage, the post-stroke neuroinflammatory response, which leads to further neuronal cell loss over an extended period, presents a clear therapeutic target to improve outcomes and reduce morbidity from stroke.

In this review, we describe the current state of our understanding of neuroinflammation and its relationship to cerebral ischemia. First, we outline the organization of the immune system in the brain and its response to stroke. Next, we discuss the involvement of different components of the acute and adaptive immune response in thromboinflammation. We then describe several neuroimaging techniques that can provide a real-time, individualized picture of the immune response in a clinical setting and its implications. Finally, we review ongoing clinical trials, translational research, and possible future directions for the field.

## Neuroinflammation in acute cerebral ischemia

This section provides a broad overview of the main cell types and timelines of the neuroinflammatory response after an ischemic stroke. Each of these in turn is then discussed further in their respective sections.

### Immune system's role in neuroinflammation

Following an ischemic stroke, the immune system is critically involved in the neuroinflammatory response. The initial damage from the stroke releases DAMPs (damage-associated molecular patterns), which activate various components of the innate immune system, such as neutrophils, macrophages, and microglia (14, 15). This activation triggers the complement system and toll-like receptors, intensifying the inflammatory response. Concurrently, the adaptive immune system is engaged, involving different lymphocyte populations including T cells, B cells, and regulatory T cells, as well as antigen-specific responses (14, 16, 17). This immune activation can compromise the integrity of the blood-brain barrier (BBB), allowing immune cells and inflammatory molecules to enter the brain, further exacerbating inflammation and stroke injury (14, 18). Importantly, this response presents a wide array of possible therapeutic targets since it involves many signaling pathways over a timescale of days to weeks.

### Overview of neuroinflammation post-stroke

The inflammatory response following a stroke evolves over time, transitioning from an acute phase to a chronic phase. Acute neuroinflammation serves as an initial defense mechanism, with microglial cells, the brain's resident immune cells, clearing out dead cells and debris (19, 20). In intracerebral hemorrhage (ICH), macrophages clear red blood cells, aiding in hematoma resolution, while in ischemic stroke, microglia and macrophages might contribute to ferroptosis, a type of non programed cell death associated with iron and lipid peroxidation (21, 22). Targeting ferroptosis is a promising therapeutic strategy, though the mechanisms are still under investigation (23–25).

Two types of edema characterize the acute and chronic phases of post stroke neuroinflammation. Cytotoxic edema occurs rapidly after an ischemic stroke within minutes to hours and is caused by loss of ATP due to reduced blood flow and glucose supply. This leads to Na+/K+ pump failure and thus cellular swelling. Cytotoxic edema peaks 3–4 days post injury (26, 27). Vasogenic edema is characterized by BBB breakdown that allows water and plasma proteins to leak into the interstitial compartment. This occurs via a transcellular route and a paracellular route through tight junction disruption. Vasogenic edema can begin as early as 6 h after a stroke and peaks 6–7 days. Since vasogenic edema causes direct tissue swelling, it is the primary driver of post stroke elevations of intracranial pressure and subsequent neurologic decline. Severe cerebral edema of either variety (but more commonly vasogenic) can increase mortality rates up to 80% (26, 27).

#### Acute thromboinflammatory response

Immediately following an ischemic event, a complex thromboinflammatory cascade is initiated, involving both thrombotic and inflammatory pathways (28, 29). Neutrophils, macrophages, and T and B cells act as key thromboinflammatory mediators, modulating the neuroinflammatory response. Neutrophils, attracted by chemokines and signaling molecules from the damaged endothelium, exacerbate damage through the release of neutrophil extracellular traps (NETs), reactive oxygen species (ROS), and proteases and arrive at the site of ischemia within minutes to hours (29, 30). They also exacerbate clot formation and inflammation by providing a scaffold for platelet adhesion and activation. Neutrophil-derived proteases and ROS further damage the BBB, allowing more immune cells to infiltrate the brain and perpetuate the cycle of inflammation and thrombosis (31, 32).

Within 24–48 h, monocytes are recruited to the ischemic brain and differentiate into macrophages, which can adopt pro-inflammatory (M1) or anti-inflammatory (M2) phenotypes (33). M1 macrophages produce pro-inflammatory cytokines and contribute to tissue damage, while M2 macrophages aid in tissue repair and resolution of inflammation. The balance between these phenotypes is dynamic and influenced by the local microenvironment and signaling cues.

After 2–3 days, the adaptive immune response predominates. T cells, particularly CD4+ and CD8+ subsets, infiltrate the brain and exacerbate inflammation through direct cytotoxicity and cytokine release (34–36). Regulatory T cells (Tregs) can mitigate these effects by suppressing excessive immune responses and promoting tissue repair. T cells have been shown to persist in the injured brain tissue up to 28 days (37). B cells, although less studied, may contribute to stroke pathology by producing antibodies that enhance inflammation or by modulating T cell responses (38, 39).

The interplay between these immune cells and the thrombotic processes underscores the complexity of neuroinflammation in stroke. Therapeutic strategies that modulate these immune responses, such as targeting specific chemokines or cytokines, hold promise for reducing tissue damage and improving recovery.

#### Innate CNS immune response: microglial activation

Microglial activation occurs swiftly post-stroke. Initially, acute inflammation can be neuroprotective, enhancing immune signaling and cytokine expression like IL-1, which helps in brain repair processes (40, 41). IL-4 also plays a role in recovery and axonal regrowth. However, as acute inflammation progresses, it can also involve pro-inflammatory cytokines like TNF-α, IL-6, and IL-1β, produced by activated microglia and neurons (40). The pro-inflammatory (M1) microglia subtype tends to dominate in this phase over the anti-inflammatory (M2) phenotype (40, 42, 43).

#### Chronic neuroinflammation

Chronic neuroinflammation has been shown to worsen stroke outcomes (44, 45). Prolonged BBB disruption allows continuous infiltration of immune cells and serum proteins into the brain tissue, which leads to sustained inflammation, increased intracranial pressure, and further cell death (44, 46). Multiple mechanisms contribute to the ongoing inflammatory response, which has been shown to exacerbate the initial stroke damage (45, 47). Dysfunctional activation of microglia and astrocytes, which also respond to brain injury, contributes to this chronic inflammation. Recent studies have suggested that there may also be a significant involvement of the peripheral immune system at this stage as well including a prolonged autoimmune response against neuronal antigens, perpetuating long-term neuroinflammation (48).

#### Systemic immunodepression

Just as important as the local CNS inflammatory response, is the systemic immunodepression that occurs (49, 50). Critically ill stroke patients often develop respiratory or genitourinary infections which significantly contribute to the in-hospital morbidity and mortality due to systemic lymphopenia and dysfunctional innate immune cells (49). These infections may also increase the chance of developing a chronic maladaptive inflammatory state which has been linked to poorer long term neurologic outcomes (49–51).

## Thromboinflammation

**Figure 1:**
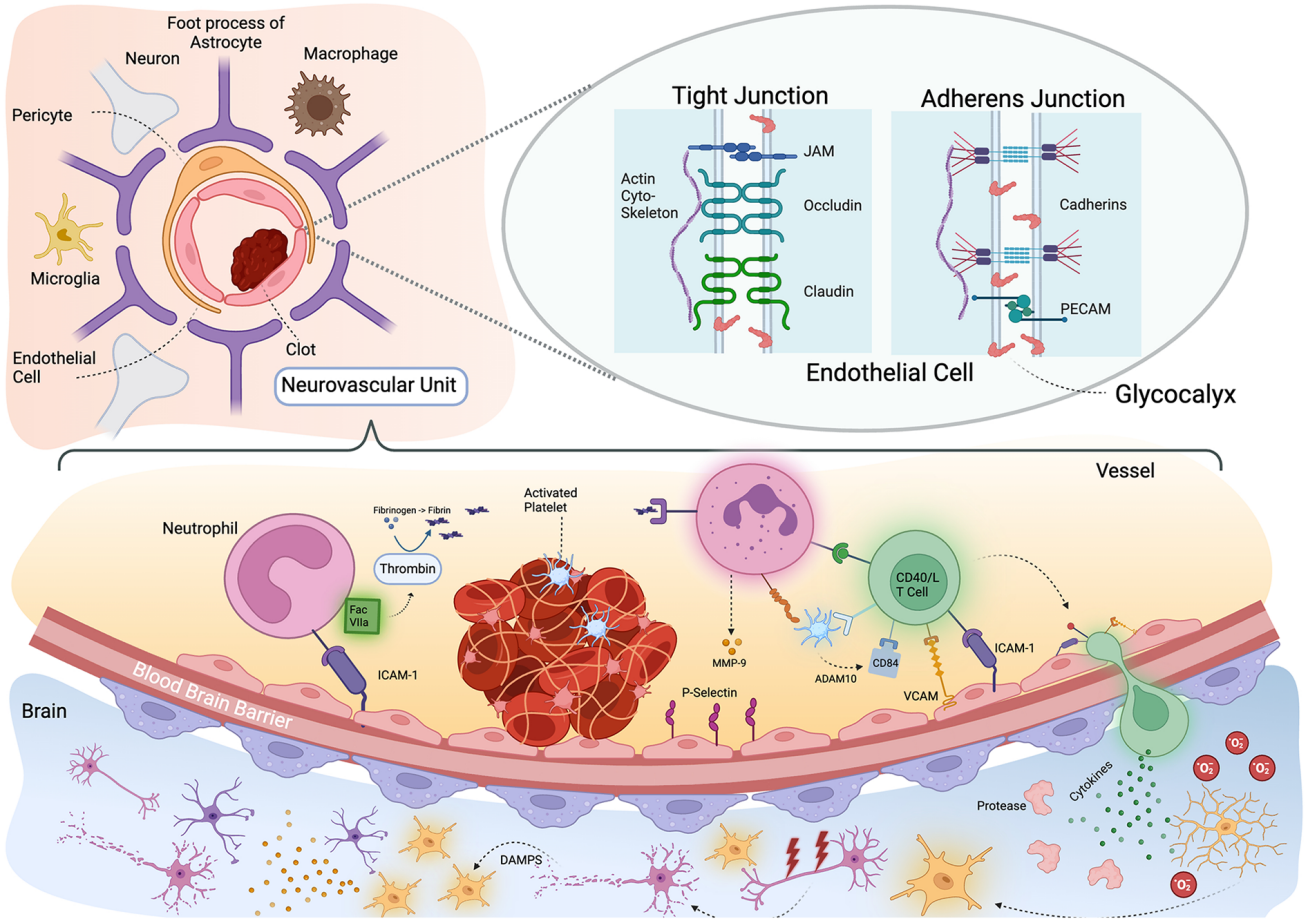
Thromboinflammation and the neurovascular unit. Schematic representation of neurovascular unit and thromboinflammation after stroke. Upper left: the neurovascular unit is comprised of the vascular endothelium, pericyte and foot processes of astrocytes. There are also associated neuronal processes and resident microglia and macrophages. Upper right: Zoomed in view on a tight junction between endothelial cells which has several subtypes. On the left shows is a tight junction where Occludin, Claudin and JAM proteins adhere to one another across the cell membrane and attach to the actin cytoskeleton. Bottom pane: an activated platelet thrombus triggers an immunologic cascade. Neutrophils respond initially with their binding stabilized by ICAM-1 and associates with Factor VIIa which catalyzes the formation of fibrin from fibrinogen. The fibrin fragments triggers further neutrophil activation and CD40/L T cells are recruited. ADAM10 and activated platelets release CD84 and the T cell migrates through the blood brain barrier. In the brain parenchyma, the T cells release cytokines, activating proteases and microglia (many of which may already be responding to the death of neurons from hypoxia) to release oxygen free radicles which damage neurons. The resulting Damage Associated Molecular Patterns (DAMPs) further activate more microglial leading to a maladaptive immune response. Created with BioRender.com.

Thromboinflammation is a pivotal mechanism contributing to the pathophysiology of ischemic stroke and involves both the inflammatory and thrombotic pathway. Research has focused on this pathway as it involves many specific and well-studied molecules and signaling cascades, which could serve as therapeutic targets. There are three general components of this system. First the initial platelet-based thrombus, second the pro-coagulopathic and pro-inflammatory contact-kinin system, and third the delayed adaptive immune response (Figure 1).

### Platelet activation and stroke

Current research underscores the importance of platelet adhesion and early activation in mediating thromboinflammatory damage post-stroke, primarily through interactions involving glycoprotein (GP) VI and integrin α_2_β_1_ with collagen, and GPIbα with von Willebrand factor (VWF) (31, 52). These interactions facilitate platelet adhesion, activation, and aggregation, crucial for thrombus formation. Notably, VWF's role in stroke is evidenced by studies showing that VWF deficiency leads to reduced ischemic damage and improved outcomes (53–55). Therapeutic approaches targeting the VWF-GPIbα axis, such as anti-VWF nanobodies and GPIbα antagonists, show promise in experimental models by reducing both thrombosis and inflammation without increasing bleeding risks (28, 56–58). However, these interventions have not been performed in humans yet.

Platelet activation begins with endothelial damage, which exposes subendothelial collagen and VWF. This exposure results in platelet tethering via the GPIb-V-IX complex. Subsequent firm adhesion is mediated by integrins, notably α_2_β_1_ binding to collagen and αII_b_β_3_ binding to fibrinogen (59). This interaction triggers platelet activation, characterized by shape change, degranulation, and the release of secondary mediators such as ADP and thromboxane A2, which amplify the activation process (60). Activated platelets aggregate to form a thrombus, stabilizing the initial hemostatic plug.

Activated platelets also release polyphosphates, which further activate the intrinsic coagulation pathway through FXII, which leads to thrombin generation and fibrin formation (61). Beyond their hemostatic function, platelets release pro-inflammatory cytokines and chemokines, such as IL-1_β_ and CD40l, which recruit and activate leukocytes, thereby linking thrombosis with inflammation (62, 63). Additionally, when activated to the inflammatory phenotype, platelets impair normal microvascular function, cause breakdown of microvascular barriers and can affect the extravasation of immune cells through gap junctions (29, 64, 65). These multifaceted roles of platelets highlight their central position in thromboinflammation, making them a prime target for therapeutic intervention.

### Kallikrein-kinin pathway

The kallikrein or contact-kinin pathway is, initiated by factor XII (FXII) activation, and it significantly contributes to thromboinflammation by promoting both coagulation and inflammation (66, 67). This complex of serine proteases both triggers thrombus formation and helps regulate the subsequent local response via kinin mediated signaling cascade. FXII activation leads to a cascade involving plasma kallikrein and bradykinin release, which exacerbates BBB permeability and inflammation, worsening the risk of subsequent hemorrhage (68, 69). Inhibition of this pathway, either genetically or pharmacologically, has been shown to reduce stroke severity in animal MCA occlusion mouse models, highlighting its potential as a therapeutic target. For instance, blocking plasma kallikrein or bradykinin receptors mitigates BBB disruption, inflammation, and thrombus formation, which suggests these interventions may provide neuroprotective effects without impairing hemostasis.

Upon vascular injury, FXII binds to exposed endothelial surfaces and becomes activated to FXIIa. This initiates the kallikrein-kinin system (KKS), where FXIIa converts plasma prekallikrein to kallikrein. Kallikrein then cleaves high-molecular-weight kininogen (HMWK) to release bradykinin, a potent vasodilator and inflammatory mediator. Bradykinin binds to its receptors, B1 and B2, on endothelial cells, which increases vascular permeability and promotes the recruitment of immune cells.

This pathway also interacts with the complement system, which is another critical component of the inflammatory response. FXIIa can activate the complement cascade, which leads to the production of anaphylatoxins such as C3a and C5a that further recruit and activate leukocytes. This crosstalk between the coagulation and complement systems amplifies the inflammatory response and contributes to tissue damage and edema in the ischemic brain.

Therapeutic interventions targeting the kallikrein-kinin system (KKS), such as FXII inhibitors, kallikrein inhibitors, and bradykinin receptor antagonists, have demonstrated substantial promise in preclinical MCA occlusion mouse models. For instance, FXII inhibitors like garadacimab have been shown to reduce thrombus formation and limit inflammation, resulting in decreased infarct sizes. Kallikrein inhibitors, such as lanadelumab, effectively block the conversion of prekallikrein to kallikrein, thereby diminishing the production of bradykinin and subsequent inflammatory responses. Bradykinin receptor antagonists, including icatibant, prevent bradykinin from binding to its receptors B1 and B2, which helps to stabilize the BBB and reduce cerebral edema. A phase 1 clinical trial ReMEDy1 (NCT03290560), testing DM199 a recombinant form of human tissue kallikrein-1 (KLK1) showed promising initial results in reducing the rate of secondary strokes. A follow up phase 2/3 trial ReMEDy2 (NCT05065216) is ongoing, though it was paused for nearly 2 years due to reports of three adverse events regarding hypotension on drug administration (70–72).

These treatments collectively reduce inflammation and stabilize the BBB, which lead to smaller infarct sizes and better overall outcomes in animal models. Importantly, these therapeutic agents have not been associated with a significant increase in bleeding risk, and they are attractive candidates for further clinical development. Their efficacy in managing thromboinflammatory processes in ischemic stroke presents a promising avenue for improving patient care and reducing the long-term impacts of stroke.

### Adaptive immune system

The adaptive immune system, particularly T cells, plays a critical role in stroke-induced thromboinflammation. Studies have demonstrated that T cell infiltration into the ischemic brain exacerbates neuronal damage through interactions with platelets and endothelial cells (35). Regulatory T cells (Tregs) and other subsets of T cells modulate this inflammatory response, with evidence suggesting that their absence can either worsen or ameliorate stroke outcomes depending on the context (73, 74). Current research is exploring the modulation of T cell activity as a therapeutic strategy, with promising results from interventions targeting T cell adhesion molecules and cytokine signaling pathways (75).

After a stroke, the damaged BBB allows immune cells to infiltrate the brain parenchyma (76). CD4+ and CD8+ T cells, which are normally involved in immune surveillance, become pathogenic in the context of ischemic injury. CD4+ T cells contribute to inflammation by releasing pro-inflammatory cytokines such as IFN-γ and TNF-α, which exacerbate neuronal damage and promote further immune cell recruitment (77). CD8+ T cells, on the other hand, directly kill neurons through perforin and granzyme B, compounds typically used to eliminate virus-infected cells (45, 77).

Regulatory T cells (Tregs) play a dual role in stroke. In the acute phase, Tregs can limit excessive inflammation and tissue damage through the release of anti-inflammatory cytokines like IL-10 and TGF-β (78). However, in some contexts, Tregs may also inhibit beneficial immune responses necessary for tissue repair and clearance of dead cells (79). The timing and context of Treg activity are therefore critical in determining their overall impact on stroke outcomes.

The role of B cells in stroke-related thromboinflammation has been less extensively studied compared to T cells, but emerging evidence suggests they play important roles. Recent research indicates that B cells may have both protective and pathogenic roles in stroke. Regulatory B cells producing IL-10 have been shown to limit CNS inflammation and reduce infarct volume in experimental stroke models (80). However, B cells can also contribute to thromboinflammation by producing autoantibodies against brain antigens exposed after BBB disruption (81). The net effect of B cells likely depends on the specific subsets involved and the timing relative to stroke onset. More research is needed to fully elucidate the diverse functions of B cells in stroke pathophysiology.

Therapies aimed at modulating T cell responses include monoclonal antibodies targeting adhesion molecules (such as ICAM-1 and VCAM-1) that facilitate T cell entry into the brain, as well as cytokine inhibitors that block the action of pro-inflammatory cytokines (82). Early clinical trials with these approaches have shown potential in reducing inflammation and improving neurological outcomes, although more research is needed to refine these strategies (83–85).

### Thromboinflammation post reperfusion therapy

Thromboinflammation post-reperfusion in ischemic stroke involves complex interactions between cellular and humoral immune mediators. Reperfusion triggers an influx of neutrophils, monocytes/macrophages, and T cells, which peak at different time points and contribute to both protective and damaging effects (86, 87). This process in the CNS differs from peripheral thromboinflammation due to the presence of the BBB and resident microglia (88). The repercussions of reperfusion on a leaky BBB include increased permeability to both small and large molecules, facilitating the entry of inflammatory mediators and potentially exacerbating tissue damage.

The von Willebrand factor (VWF) A1 domain plays a crucial role in mediating the inflammatory response during cerebral ischemia/reperfusion by recruiting inflammatory monocytes, neutrophils, and T cells (56). This recruitment process contributes to the thromboinflammatory cascade, which involves highly interconnected thrombotic and neuroinflammatory signatures. The central-peripheral immune communication after ischemic stroke is facilitated by various mechanisms, including the release of damage-associated molecular patterns (DAMPs) into the periphery and the transmission of inflammatory signals from the brain to the spleen via the sympathetic nervous system and hypothalamic-pituitary-adrenal axis (89, 90).

BBB disruption, a major hallmark of stroke, is initiated by ischemia and continues to deteriorate with sustained hypoperfusion. This breakdown occurs partially through the overexpression of matrix metalloproteinases (MMPs), which can degrade the endothelial glycocalyx, a known mechanotransducer (8). The sustained increase in BBB permeability is likely due to a neuroinflammatory response, which, combined with other consequences such as brain edema, contributes to longer-term permanent loss of neurological function (91).

### Clinical implications and future directions

Antiplatelet agents such as clopidogrel or ticagrelor are already in widespread clinical use and target this pathway (92, 93). Other agents remain under investigation. For example, GPVI antagonists are being tested for their efficacy in reducing stroke severity and improving outcomes (94). Additionally, the potential repurposing of drugs used in other inflammatory conditions, such as multiple sclerosis, for stroke treatment is being investigated (95, 96). Overall, understanding the intricate interplay between thrombosis and inflammation in stroke is paving the way for novel therapeutic approaches that could significantly enhance patient outcomes by addressing the underlying thromboinflammatory mechanisms.

Targeting the Kallikren-Kinin pathway is another promising approach. FXII inhibitors, such as garadacimab, have shown efficacy in reducing thrombus formation and inflammation in animal models (97). A clinical trial of this agent reduced the angioedema attacks in patients with hereditary angioedema with dysfunction of factor XII (98, 99). This would be an interesting agent to test in the stroke population. Similarly, bradykinin receptor antagonists, such as icatibant, have demonstrated protective effects by reducing BBB permeability and edema (100).

Anti-inflammatory strategies are also being explored, including the use of Toll-like receptor 4 (TLR4) antagonists and interleukin-1β (IL-1β) inhibitors (47, 101). Early clinical trials have shown promising results, with agents like ApTOLL and Canakinumab demonstrating potential in reducing inflammation and improving recovery (102, 103). Fingolimod, a sphingosine-1-phosphate receptor modulator used in multiple sclerosis, has also shown potential in reducing infarct size and improving outcomes in stroke patients (95, 96, 104).

The complex interplay between thrombotic and inflammatory processes in stroke highlights the need for multifaceted therapeutic approaches, but also offers promise because of the many potential therapeutic targets. Combination therapies that target both thrombosis and inflammation may offer the best outcomes. Ongoing research and clinical trials will be critical in identifying the most effective strategies and translating these findings into clinical practice.

## Secondary cell death in stroke

Secondary cell death following ischemic stroke significantly contributes to the overall damage and neurological deficits experienced by patients. This process is not only driven by the initial ischemic insult but is also profoundly influenced by the body's immune response, particularly the adaptive immune system.

### Mechanisms of secondary cell death

**Figure 2:**
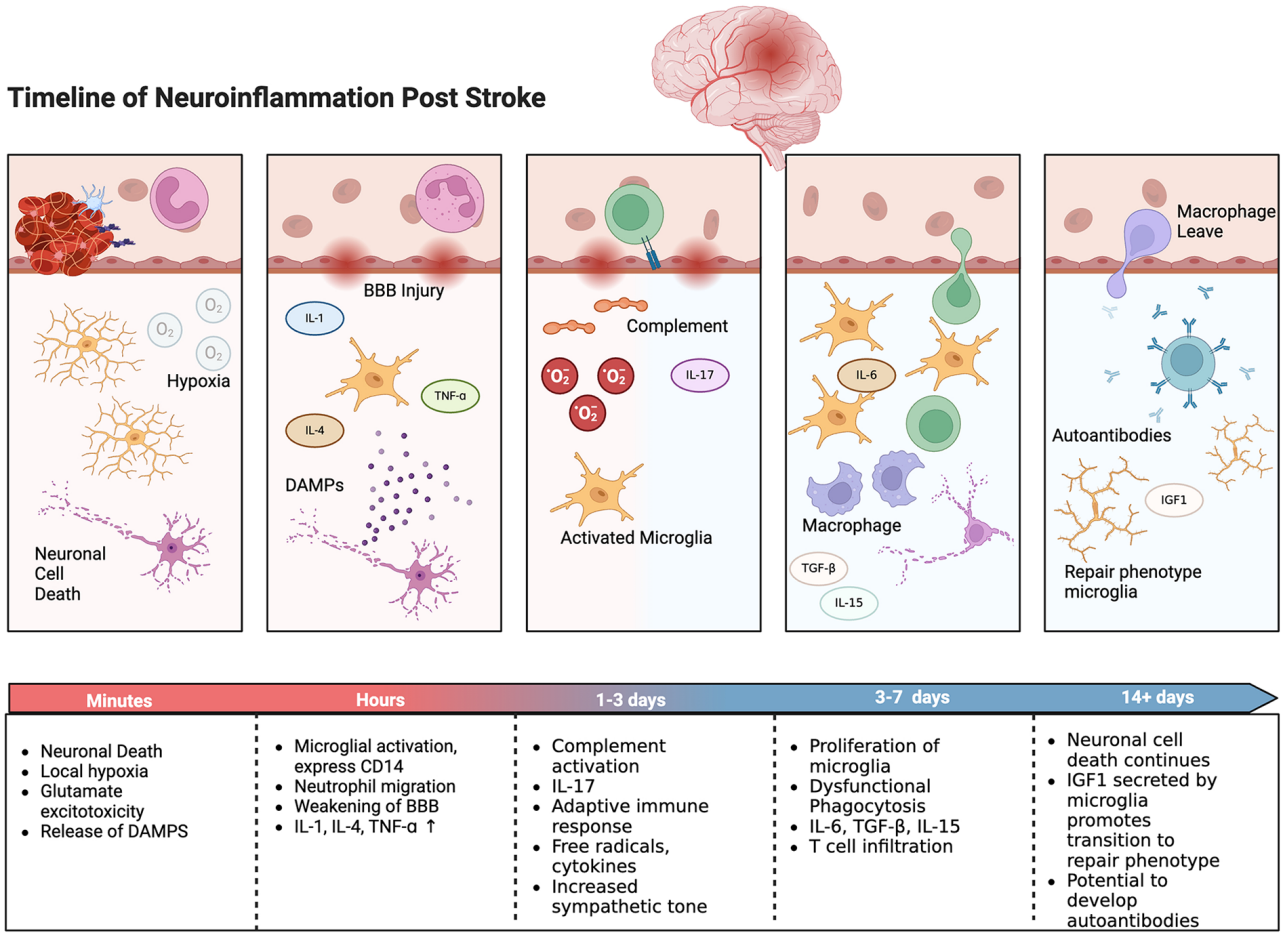
Timeline of neuroinflammatory response with selected inflammatory cells and events shown. Created with BioRender.com.

Ischemic stroke disrupts the BBB and triggers a robust inflammatory response. The primary ischemic injury sets off a chain reaction involving excitotoxicity, oxidative stress, and the release of damage-associated molecular patterns (DAMPs), which further exacerbate the injury (Figure 2). Excessive release of glutamate leads to overactivation of NMDA receptors, which in turn results in calcium overload and neuronal injury. Reactive oxygen species (ROS) generated during ischemia/reperfusion damage cellular components, leading to lipid peroxidation, protein oxidation, and DNA damage. The ischemic insult elevates levels of DAMPs, activating microglia and astrocytes and attracting peripheral immune cells to the injury site (105). This immune response, while initially protective, often becomes dysregulated, leading to further neuronal damage.

### Acute immune response

Neutrophils are among the first peripheral immune cells to infiltrate the ischemic brain, which typically peaks within the first 3 days post-stroke. These cells exacerbate tissue damage through the release of proteolytic enzymes, ROS, and pro-inflammatory cytokines (106). Elevated levels of matrix metalloproteinases (MMPs), particularly MMP-9, are associated with BBB breakdown and hemorrhagic transformation (107). Neutrophil depletion or inhibition of MMP activity has been shown to reduce BBB permeability and infarct size, which suggests that neutrophil inhibition may be an attractive therapy for acuter ischemic stroke (108). Interestingly, in animal models there is evidence that neutrophils from the bone marrow of the skull can utilize microvascular channels to migrate to the neuroinflammatory site, potentially indicating that there may be a role for blockading this local bone marrow effect (109).

Monocytes are recruited to the ischemic brain shortly after neutrophils, where they differentiate into macrophages. These cells play a dual role in stroke pathology. In the early stages, macrophages contribute to inflammation and tissue damage by producing pro-inflammatory cytokines such as TNF-α and IL-1β (90). However, during the later stages, macrophages can adopt an anti-inflammatory phenotype, promoting tissue repair and remodeling by secreting growth factors and anti-inflammatory cytokines like IL-10 (90, 110).

### The role of microglia in secondary cell death

Microglia, the resident immune cells of the central nervous system (CNS), play a crucial role in the immediate and long-term response to ischemic stroke. Following the ischemic insult, microglia are rapidly activated, migrating to the site of injury. This activation is essential for debris clearance and initial neuroprotection; however, prolonged or excessive activation can lead to exacerbated neuroinflammation and secondary cell death (20, 111).

Microglia exhibit a range of phenotypes post-stroke, including both proinflammatory (M1) and anti-inflammatory (M2) subtypes (112). This categorization has fallen out of favor as it does not capture the complex range of responses, however it is still a useful structural framework to conceptualize the response. The M1 phenotype is typically associated with the production of pro-inflammatory cytokines, such as TNF-α, IL-1β, and IL-6, and is linked to neurotoxicity and the perpetuation of inflammation (113, 114). On the other hand, the M2 phenotype is associated with the release of anti-inflammatory cytokines, such as IL-10 and TGF-β, and is involved in tissue repair and neuroprotection (114).

The differentiation between these subtypes is influenced by various signals in the microenvironment (115). For example, interferon-gamma (IFN-γ) and lipopolysaccharide (LPS) can drive microglia towards the M1 phenotype, while IL-4 and IL-13 promote the M2 phenotype (20, 114). Single cell RNA studies have indicated that there can be many microglial cell subsets within an area of ischemia neural tissue (111, 113, 116, 117).

The interplay between microglia and adaptive immune cells is critical in determining the extent of secondary cell death, and this relationship is poorly understood in stroke patients. For instance, microglia can present antigens to T cells, influencing their activation and differentiation (111, 114). This interaction can either amplify the inflammatory response or contribute to its resolution, depending on the context and the signals involved.

This plasticity of microglial responses underscores their potential as therapeutic targets in managing neuroinflammation and secondary cell death post-stroke.

### Local adaptive immune system and secondary cell death

The adaptive immune system also plays a critical role in the progression of secondary cell death following stroke. The activation of the adaptive immune response involves T cells, B cells, and regulatory T cells (Tregs), each contributing to the inflammatory milieu post-stroke (86, 87, 118). CD4+ and CD8+ T cells infiltrate the CNS following BBB disruption. CD4+ T cells can differentiate into various subsets, including Th1 and Th17 cells, which produce pro-inflammatory cytokines that exacerbate neuroinflammation and tissue damage (35). CD8+ T cells can directly induce neuronal apoptosis through cytotoxic mechanisms. Regulatory T cells (Tregs), on the other hand, have a neuroprotective role by secreting anti-inflammatory cytokines such as IL-10 and TGF-β, which can suppress the activation of harmful immune responses and promote tissue repair (34, 119).

B cells and the antibodies they produce may also contribute to post-stroke inflammation, though evidence suggest they are less important than T cells (38, 120). Autoantibodies targeting CNS antigens can exacerbate neuronal damage and inflammation. However, B cells can also have regulatory roles, producing IL-10 and other factors that help modulate the immune response and promote resolution of inflammation (38).

### Chronic neuroinflammation and its impact

Chronic inflammation following stroke leads to sustained injury and impaired recovery. Persistent activation of microglia and astrocytes, along with continuous recruitment of peripheral immune cells, creates a hostile environment for neuronal survival and regeneration (48). The prolonged inflammatory response damages the BBB and allows further infiltration of immune cells and perpetuates a cycle of injury. Chronic inflammation is associated with ongoing neuronal loss and white matter damage, which contributes to cognitive decline and functional deficits (121, 122). Secondary neurodegeneration following stroke is characterized by progressive neuronal death and gliosis in regions connected to the primary lesion site, such as the thalamus, hippocampus, and corpus callosum (123). Biomarkers such as neurofilament light chain (Nf-L), tau protein, and S100b reflect the extent of brain injury and are associated with secondary neurodegeneration and correlate with worse functional outcomes post stroke (124, 125).

### Interactions between immune cells in stroke-related neuroinflammation

It is essential to highlight how the complex interplay between different immune cell types plays a crucial role in shaping the neuroinflammatory response after ischemic stroke. While more challenging to study than single cell type effects, this network of interactions better represents the physiologic environment and involves both resident and infiltrating immune cells, each contributing to the progression and resolution of inflammation in unique ways.

#### Microglia-T cell interactions

Microglia, the brain's resident immune cells, interact closely with infiltrating T cells to modulate the inflammatory response. After stroke, activated microglia can present antigens to T cells, influencing their differentiation and function (43). T cell subsets, in turn, have at least three distinct effects on microglial activation: (1) Th1 cells promote a pro-inflammatory microglial phenotype by secreting IFN-γ, which induces the expression of pro-inflammatory cytokines like TNF-α and IL-1β (43, 126). (2) Regulatory T cells (Tregs) encourage an anti-inflammatory microglial phenotype by producing IL-10 and TGF-β, which can suppress excessive inflammation and promote tissue repair (126, 127). (3) A recent study demonstrated that early microglial activation in response to stroke is differentially regulated by T cell subpopulations, with Th1 cells inducing type I interferon signaling in microglia and Tregs promoting microglial genes associated with chemotaxis (126).

#### Astrocyte-immune cell crosstalk

Astrocytes, another key glial cell type, engage in bidirectional communication with various immune cells though several mechanisms. First, activated astrocytes can produce chemokines that attract T cells, neutrophils, and monocytes to the site of injury (128). Second, CD8+ T cells and NK cells have been observed to co-localize with astrocytes in peri-infarct regions 24 h after stroke, suggesting direct cellular interactions (129). Lastly, astrocyte interactions with Tregs have been reported to limit CNS inflammation, highlighting the complex role of these glial cells in modulating the immune response (130).

#### Neutrophil-endothelial cell interactions

As discussed, neutrophils are among the first peripheral immune cells to respond to stroke. Within hours of stroke onset, neutrophils interact with activated endothelial cells through adhesion molecules like ICAM-1, P-selectin, and E-selectin. This interaction leads to neutrophil infiltration into the brain parenchyma, where they release inflammatory mediators and contribute to BBB breakdown (48, 131).

#### B cell interactions with the thromboinflammatory cascade

While less studied than T cells, B cells also play a role in the neuroinflammatory response to stroke. Regulatory B cells producing IL-10 have been shown to limit CNS inflammation and reduce infarct volume in experimental stroke models. However, B cells can also contribute to thromboinflammation by producing autoantibodies against brain antigens exposed after BBB disruption (127).

Overall, the intricate network of interactions between various immune cell types in stroke-related neuroinflammation involves a delicate balance of pro-inflammatory and anti-inflammatory signals. Understanding these interactions is crucial for developing targeted immunomodulatory therapies for stroke. Future research should focus on elucidating the temporal dynamics of these interactions and identifying key molecular mediators that could serve as therapeutic targets.

### Potential therapeutic interventions

Given the significant role of the adaptive immune system in secondary cell death, targeting these immune responses presents a promising therapeutic strategy. Modulating microglial activation, promoting Treg function, and inhibiting pro-inflammatory T cell responses are potential approaches to reduce secondary damage and improve outcomes following stroke. Pharmacological agents that can skew microglia towards a neuroprotective phenotype, such as minocycline or laquinimod, show promise in reducing neuroinflammation (132, 133). Additionally, therapies aimed at enhancing Treg function, such as low-dose IL-2 or Treg adoptive transfer, could provide neuroprotection by modulating the adaptive immune response (134, 135). The use of stem cell therapy has also been explored to modulate the immune response and promote recovery. Mesenchymal stem cells (MSCs), for example, can secrete factors that promote the M2 phenotype in microglia and enhance Treg function, providing a multifaceted approach to reducing inflammation and promoting tissue repair (136–138).

Secondary cell death in stroke is a complex process driven by both the initial ischemic insult and the subsequent immune response. The adaptive immune system, through its various cellular components and interactions with microglia, plays a pivotal role in this process. Understanding these mechanisms provides valuable insights into potential therapeutic strategies aimed at reducing secondary damage and promoting neurological recovery following stroke. Further research and clinical trials are necessary to translate these findings into effective treatments for stroke patients.

## Blood brain barrier and peripheral neuroinflammation

**Figure 3:**
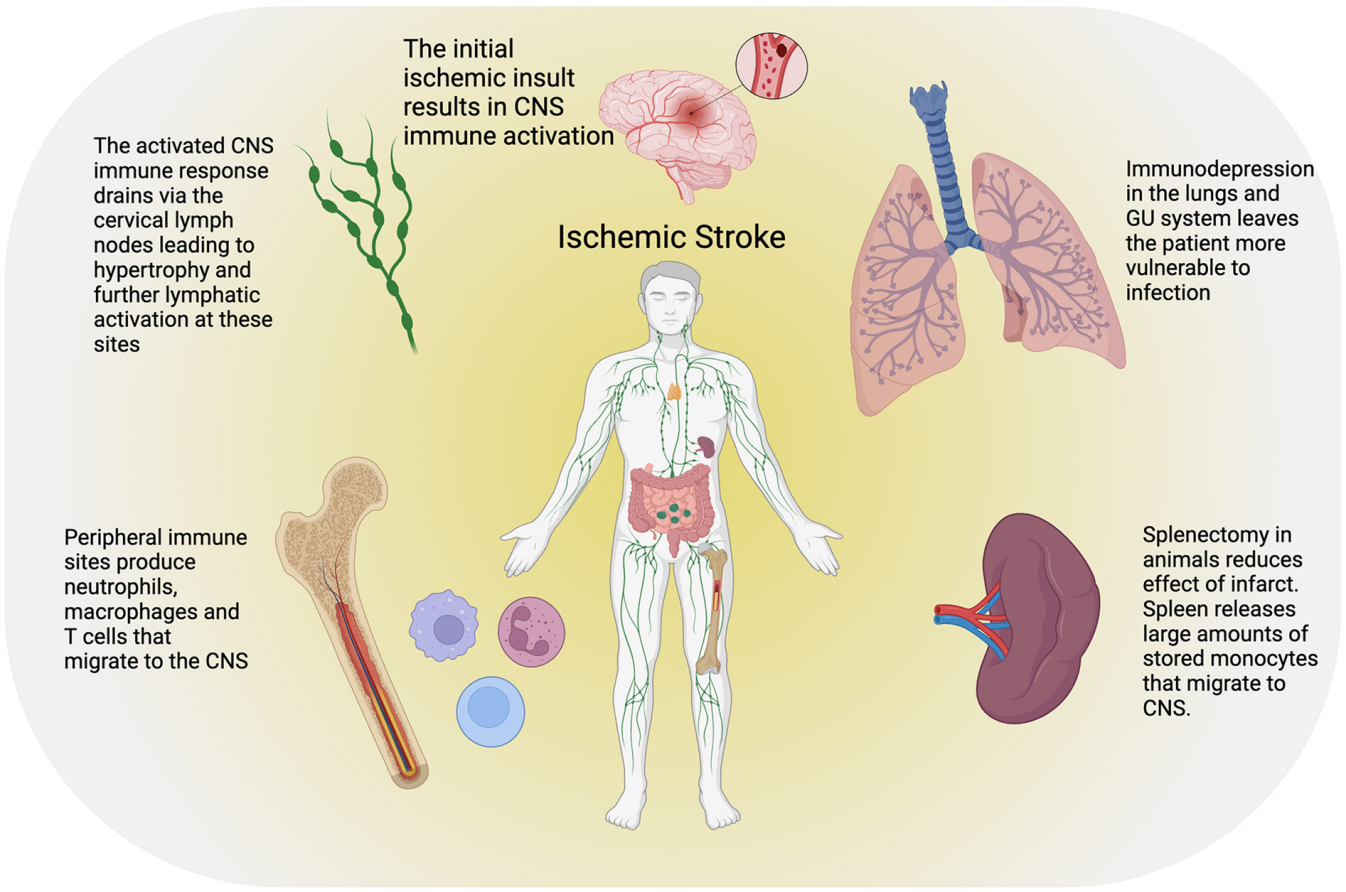
Ischemic stroke results in a complex peripheral immune response as well as paradoxically an increased risk of infection due to global immunodepression. CNS immune cells and inflammatory signals drain to the cervical lymph nodes causing cervical lymphadenopathy and subsequent increased hematopoiesis in the axial bone marrow. The spleen also serves as a ready store of macrophages that are rapidly mobilized after stroke and can migrate to the site of injury. Created with BioRender.com.

The peripheral immune system plays a critical role in the response to ischemic stroke, and it likely impacts both acute injury and long-term recovery (Figure 3). Neuroinflammation, characterized by the activation and infiltration of peripheral immune cells, contributes to BBB disruption, neuronal injury, and secondary brain damage. Understanding the mechanisms by which peripheral immune cells influence stroke pathology is crucial for developing targeted therapeutic strategies.

### Blood-brain barrier disruption

The BBB is a highly selective structural and functional barrier that regulates the exchange of substances between the blood and the central nervous system (CNS). It is composed of endothelial cells, pericytes, astrocytes, neurons, microglia, and perivascular macrophages, collectively known as the neurovascular unit. This unit maintains CNS homeostasis and protects the brain from harmful substances and pathogens. However, ischemic stroke induces significant BBB disruption, leading to increased permeability and the infiltration of peripheral immune cells (45).

Three key processes combine to disrupt the BBB: oxidative stress, Matrix Metalloproteinases (MMPs) and Inflammatory Cytokines. Following ischemic stroke, excessive production of reactive oxygen species (ROS) damages endothelial cells and other components of the neurovascular unit (Figure 1). This oxidative stress compromises the structural integrity of the BBB, contributing to its breakdown. Antioxidant mechanisms are impaired, further exacerbating BBB damage. MMPs, particularly MMP-2 and MMP-9, are rapidly upregulated in response to ischemic injury (139–142). These enzymes degrade extracellular matrix components and tight junction proteins, leading to increased BBB permeability. Elevated MMP activity is associated with vasogenic edema and hemorrhagic transformation, highlighting the detrimental role of these proteases in BBB disruption (141, 143).

Pro-inflammatory cytokines such as tumor necrosis factor-alpha (TNF-α), interleukin-1 beta (IL-1β), and interleukin-6 (IL-6) are released by activated microglia and infiltrating immune cells (22, 142). These cytokines induce the expression of adhesion molecules on endothelial cells, promoting leukocyte adhesion and transmigration across the BBB. The resulting infiltration of immune cells further amplifies inflammation and BBB breakdown (144).

The integrity of the BBB is crucial for determining the extent of brain damage and the clinical outcome of stroke patients. Imaging techniques such as dynamic contrast-enhanced MRI and CT scans are used to assess BBB permeability *in vivo* and are discussed separately. Increased BBB permeability is associated with worse neurological outcomes and a higher risk of hemorrhagic transformation following thrombolytic therapy (145).

#### Angiogenesis and neuroinflammation

Additionally, in addition to BBB disruption, neuroinflammation can both promote and inhibit angiogenesis after stroke, depending on the timing and specific mediators involved. In the acute phase, pro-inflammatory factors like TNF-α and IL-1β can stimulate angiogenesis by upregulating VEGF expression (146). However, chronic inflammation may impair angiogenesis and vascular remodeling.

Immune cells play crucial roles in post-stroke angiogenesis. For example, M2 polarized macrophages promote angiogenesis by secreting growth factors like VEGF and bFGF (147). T cells, particularly Th2 and regulatory T cells, can also support angiogenesis through the production of IL-4 and IL-10 (31).

Conversely, newly formed blood vessels influence the neuroinflammatory response by facilitating the infiltration of peripheral immune cells into the brain parenchyma. This creates a feedback loop between angiogenesis and neuroinflammation that shapes stroke recovery (148).

### Spleen as a key peripheral immunologic site in stroke

The spleen, a crucial lymphatic organ, mounts an immune response post-stroke that worsens the pathology. It acts as a repository for immune cells, deploying monocytes to the injury site (90). Studies have shown that splenectomy (removal of the spleen) prior to stroke significantly reduces cerebral infarction and the presence of monocytes in the brain, highlighting the spleen's role in stroke-induced inflammation.

The spleen serves as a reservoir for immune cells, including monocytes, which are rapidly deployed to sites of injury. After a stroke, monocytes from the spleen migrate to the brain, where they differentiate into macrophages and exacerbate inflammation. Post-stroke, the spleen undergoes contraction, releasing stored monocytes into the bloodstream which differentiate into macrophages and dendritic cells. This rapid deployment of immune cells to the brain can worsen the inflammatory response and contribute to secondary cell death.

Research involving splenectomy in animal models has demonstrated mixed results. Splenectomy prior to permanent middle cerebral artery occlusion (MCAO) significantly decreases cerebral infarction (149). However, in cases of transient MCAO, splenectomy reduces monocyte infiltration in the brain but does not significantly alter cerebral infarction, suggesting the involvement of different monocyte subsets in stroke pathology (90).

The spleen contains heterogeneous populations of monocytes, particularly the pro-inflammatory Ly6C^hi^ and anti-inflammatory Ly6C^lo^ subsets (150–153). Stroke induces the deployment of both subsets from the spleen to the brain, where they contribute to the inflammatory response. Ly6C^hi^ monocytes express high levels of the chemokine receptor CCR2 and are recruited to the injury site by monocyte chemoattractant protein-1 (MCP-1) (150). Their rapid deployment to the brain post-stroke exacerbates inflammation. Ly6C^lo^ monocytes are predominately anti-inflammatory monocytes express high levels of the receptor CX3CR1 and are recruited to normal tissues for tissue repair and maintenance (154). Their deployment to the brain post-stroke is more sustained and contributes to the resolution of inflammation.

#### Brain-spleen inflammatory coupling

The concept of brain-spleen inflammatory coupling highlights the systemic nature of stroke-induced inflammation. Brain injury triggers autonomic responses that lead to the release of proinflammatory cytokines from splenic macrophages. This interaction exacerbates central inflammation and contributes to the overall pathology of stroke (90, 149).

Intravenous administration of stem cells in stroke models has shown that these cells preferentially migrate to the spleen and reduce systemic inflammation (138, 155). This observation suggests that targeting the spleen with stem cell therapy could be an effective strategy to mitigate peripheral inflammation and improve stroke outcomes. Peripheral administration of stem cells offers a minimally invasive approach that bypasses the need to cross the BBB. This method allows for repeated treatments to address chronic inflammation, making it a practical and feasible option in clinical settings.

Developing therapies that selectively ablate specific subsets of splenic monocytes could provide a more refined approach to reducing the detrimental effects of peripheral immune responses in stroke. The autonomic regulation of splenic immune responses through cholinergic input opens new avenues for therapeutic interventions. Modulating sympathetic and parasympathetic tones could help balance proinflammatory and anti-inflammatory responses, reducing the overall inflammatory burden in stroke patients.

### Cervical lymph nodes post stroke

Another aspect of the peripheral immune response to stroke involves cervical lymph node hypertrophy, likely from CNS drainage coupled with the large-scale release of vascular endothelial growth factor C (VEGF-C) (156, 157). Blocking this receptor reduces endothelial inflammation in the cervical lymphatic system, decreases macrophage activation and has been shown in mice studies (male C57BL6 mice) to lessen brain tissue infarction (156). Brain to cervical lymph node signaling has also been shown to modulate CD4 and CD11b T cell neuronal infiltration and subsequent secondary brain damage after injury in animal models (158). In animal experiments with a cervical lymphadenectomy can partial ameliorate some of the peripheral immune system's negative affects post stroke (156, 157).

## Immunodepression and effect on recovery

Stroke-induced immunodepression is a significant factor that contributes to poor outcomes in stroke patients. Immediately following a stroke, a neuroinflammatory process begins in the brain, which simultaneously triggers systemic immunodepression through the excessive activation of the autonomic nervous system. This immunodepression manifests as lymphopenia and dysfunctional innate and adaptive immune cells, significantly impairing antibacterial defenses and rendering stroke patients highly susceptible to infections (159).

Infections occur in up to 30% of stroke patients, with pneumonia and urinary tract infections (UTIs) being the most common (160–162). Stroke-associated pneumonia (SAP) and UTIs are particularly prevalent and occur in approximately 10% of patients. Among these, pneumonia has the most substantial impact, increasing the risk of unfavorable outcomes and mortality (162). The STROKE-IFN trial highlighted that pneumonia independently increased the odds of an unfavorable outcome at 3 months post-stroke [odds ratio (OR), 9.64 (5.06–18.42)] (163). However, neither the STROKE-IFN trial, nor the similar PASS trial demonstrated any benefit to preventative antibiotics in stroke (163, 164).

Systemic infections further complicate the patient's condition by enhancing autoreactive immune responses against brain antigens such as myelin basic protein (MBP) and glial fibrillary acidic protein (GFAP), though studies have not yet found an impact on functional outcomes (165).

The susceptibility to infections is not only due to lymphopenia but also to the functional impairments of immune cells. Stroke-induced immunodepression shifts the immune response from a Th1 (pro-inflammatory) to a Th2 (anti-inflammatory) profile, weakening the body's ability to fight bacterial infections (166, 167). This shift is characterized by decreased production of pro-inflammatory cytokines like interferon-gamma (IFN-γ) and tumor necrosis factor-alpha (TNF-α), and increased levels of anti-inflammatory cytokines such as interleukin-10 (IL-10) (167).

In conclusion, stroke-induced immunodepression significantly worsens patient outcomes by increasing susceptibility to infections, which in turn exacerbate neuroinflammation and impair recovery. Understanding and mitigating this immunodepression is essential for improving the prognosis and quality of life for stroke patients.

## Imaging neuroinflammation

Several imaging modalities are available to visualize and quantify various aspects of this inflammatory response to ischemic stroke. These imaging techniques are critical for understanding the underlying mechanisms, guiding therapeutic interventions, and monitoring treatment efficacy.

Medical imaging technologies such as computed tomography (CT), magnetic resonance imaging (MRI), and positron emission tomography (PET) have significantly advanced stroke diagnostics and the study of neuroinflammation. These modalities enable the *in vivo* detection of BBB permeability, leukocyte infiltration, microglial activation, and the upregulation of cell adhesion molecules.

### Blood brain barrier permeability imaging

The disruption of the BBB is a hallmark of neuroinflammation, and it plays a critical role in the pathophysiology of various neurological conditions, including stroke. Imaging the permeability of the BBB provides insights into the extent and dynamics of this disruption. BBB permeability can be assessed using the extravasation of contrast agents or radioactive tracers, which result in image contrast enhancement (45, 168). This enhancement allows for the visualization of areas where the BBB is compromised.

Dynamic contrast-enhanced MRI (DCE-MRI) and CT (DCE-CT) scans are commonly employed techniques to quantitatively measure BBB permeability, though non-contrast techniques have also been described (169, 170). These imaging modalities facilitate the derivation of key parameters such as the extraction fraction, blood-to-brain transfer constant (K_trans), and the permeability-surface area product (PS) (171–173). These quantitative measures provide detailed information about the degree and spatial distribution of BBB disruption, offering a more precise assessment of neuroinflammatory processes.

**Figure 4:**
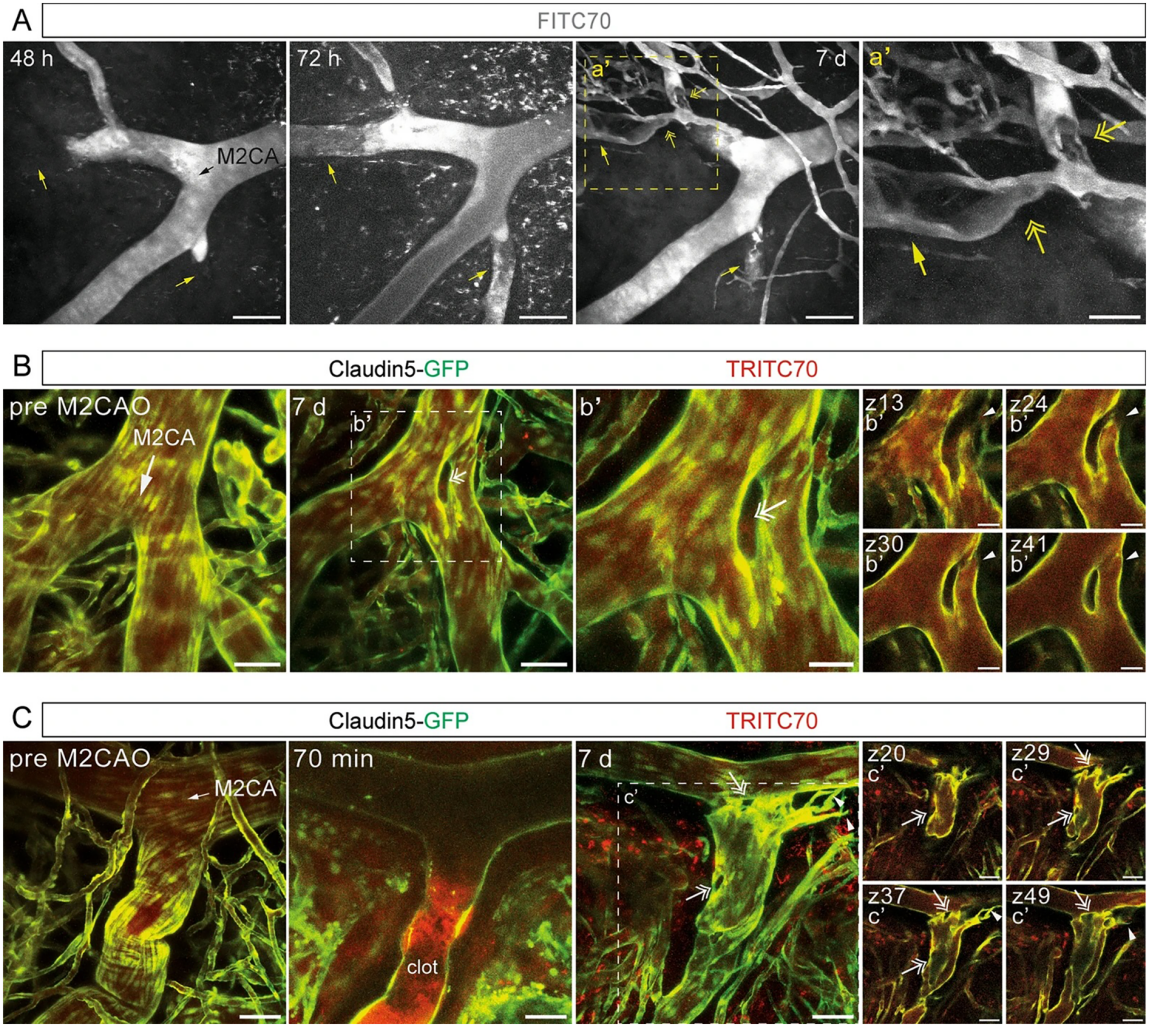
Studies in mice can provide single cell resolution of blood brain barrier (BBB) breakdown in ischemic stroke. This image demonstrates longitudinal imaging of the cerebral vasculature during the subacute and chronic phase after ischemic stroke. **(A)** Maximum intensity projections of two-photon imaged *z*-stacks of a fluorescent tracer (FITC70 signal) in an Middle Cerebral Artery (MCA) mice stroke model field of view 48 h, 72 h and 7 days post MCA occlusion, respectively. Black arrow indicates direction of blood flow; yellow arrows indicate vessels with blocked (48 h) and re-established blood flow at transition to chronic phase (72 h/7 days); yellow two-headed arrows indicate putative residual clots; scale bars 100 µm. **(B,C)** Maximum intensity projection of M2CA field of view in Claudin5-GFP reporter mice depicting endothelial cells (green) and TRITC70 signal (red) before and after MCA occlusion; (b’, c’) depict individual *z*-planes. White arrows indicate direction of blood flow; arrowheads indicate loop formed in bifurcation (b’) and angiogenic sprouting (c’); two-headed arrows indicate intussusceptive pillars; scale bars (**B**,**C**, c’) 50 µm; scale bars (b’) 25 µm. Reproduced with permission: Protzmann et al. (174).

Animal studies utilizing advanced imaging in a mouse model (Claudin5-GFP) have also offered new insights into the mechanisms of BBB disruption in ischemic stroke. Fredriksson and colleagues established a protocol for longitudinal two-photon imaging in mice, enabling the observation of BBB breach at single-vessel resolution as early as 30 min after stroke induction (174). They reveals that the disruption of BBB, along with vascular leakage, can be tracked in real time, contributing to a deeper understanding of the hemodynamic changes and vascular remodeling, such as intussusception and angiogenic sprouting, over both acute and chronic phases. Importantly, this method allows the study of vessel-specific vulnerabilities along the arteriovenous axis, which could guide the development of targeted therapeutic strategies to mitigate the long-term impacts of stroke (Figure 4).

### Leukocyte infiltration and microglial activation

Advanced MRI and PET imaging techniques have been developed to detect and quantify leukocyte infiltration and microglial activation (175, 176). For instance, the intravenous injection of ultrasmall particles of iron oxide can highlight areas of inflammation due to their uptake by circulating monocytes or phagocytosed by macrophages or microglia (45, 177, 178). PET imaging with radiolabeled ligands targeting specific inflammatory markers, such as the 18-kDa translocator protein (TSPO), provides high sensitivity and specificity for detecting microglial activation (179, 180).

PET imaging using radiotracers targeting specific inflammatory markers, such as the somatostatin receptor (SSTR) and chemokine receptors like CXCR4 and CCR2, have shown promise in detecting and monitoring neuroinflammatory processes with good specificity (181, 182).

### Cell adhesion molecules

The expression of cell adhesion molecules like VCAM-1 and ICAM-1 can be visualized using targeted molecular imaging probes. For example, MRI enhanced with iron oxide particles functionalized with antibodies against VCAM-1 has been used to detect upregulation in and around stroke lesions (183, 184). Similarly, dual-tracer PET studies have demonstrated differential time courses for markers like MMPs and TSPO, highlighting their roles in the neuroinflammatory cascade after stroke (45, 180).

### Recent advances and clinical implications

Recent studies have demonstrated the utility of advanced imaging techniques in both preclinical and clinical settings. For example, multimodal imaging combining PET and MRI has enabled detailed mapping of neuroinflammatory processes, such as the time-course of microglial activation and BBB permeability changes following stroke (178, 184). These imaging approaches not only enhance our understanding of stroke pathology but also aid in the development and monitoring of therapeutic interventions.

### Future directions, imaging neuroinflammation

While many imaging modalities have shown promise in preclinical studies, their routine clinical application remains limited. Future research should focus on developing standardized protocols for image acquisition and analysis, as well as translating these techniques into clinical practice. The development of biocompatible and biodegradable contrast agents with high specificity for human cells and epitopes will be crucial for effective clinical implementation. Additionally, multimodal and multifunctional theranostic nanoprobes that combine diagnostic and therapeutic capabilities could revolutionize the management of neuroinflammation in stroke.

## Future directions and ongoing translational trials

### Sex difference in stroke

Recent studies have illuminated significant sex differences in the immune response to stroke, revealing intricate interactions between biological sex and the immune system's behavior post-stroke. Factors such as host genetics and chromosomal sex play a critical role in shaping the host immune system and neuroimmune response to brain injury (185–187). Epidemiological data indicate that older women have poorer functional outcomes compared with men, partly due to the older age at which they experience their first stroke and the increased comorbidities seen with aging (188). This disparity is also attributed to differences in the immune response between men and women, which leads to altered inflammatory events contributing to sex differences in post-stroke recovery.

Further, evidence from preclinical studies suggests that males and females exhibit distinct activation states of both the innate and adaptive immune compartments post-stroke (188, 189). For instance, females show differentially expressed genes acutely following cardioembolic stroke, which is not observed in males (190, 191). Experimental stroke MCA occlusion mouse C57BL/6N models indicate that aged males have greater brain infiltration of neutrophils compared with age-matched females, corroborated by higher levels of neutrophil-specific cytokines such as MCP-1 and G-CSF in the circulation of aged males (192). This neutrophil infiltration may contribute to an increased incidence of hemorrhagic transformation in males (193). The study also found that females exhibit higher expression levels of Toll-like receptors (TLRs) on dendritic cells, leading to more effective antigen sensing, processing, and presentation, potentially mediated by X chromosome-encoded genes (194).

Emerging evidence suggests that there are innate sex difference in the microglia population even in the healthy adult brain. It has been observed that microglia in males are more prone to an inflammatory phenotype whereas in females the anti-inflammatory phenotype predominates (185, 195, 196). Male microglia display higher expression of interferon-stimulated genes, whereas female microglia express genes associated with development and morphogenesis, which are linked to the inhibition of the inflammatory response (196, 197). These sex-specific gene expression patterns suggest that female and male microglia inherently differ even in the healthy brain and contribute to their differential responses to ischemic stroke.

Clinical studies identified distinct biomarkers and immune responses between males and females. For example, CCL20, ICAM1, and PTGS2 were identified as sex-specific targets, with males exhibiting a stronger CD8+ T-cell response and females showing a more pronounced monocytic response (198). Additionally, sex differences in inflammatory markers such as IL-6, CRP, and S100B were observed, with these biomarkers being more elevated in female stroke patients (199). The studies also reported that females tend to have a higher risk of adverse outcomes, such as poorer recovery and increased inflammation, compared to males (185).

Collectively, these studies underscore the importance of considering sex as a biological variable in stroke research. They highlight the necessity for more sex-specific research to better understand the mechanisms underlying these differences, which could lead to more effective, tailored therapeutic strategies for both men and women post-stroke.

While the acute phase of neuroinflammation following a stroke is well-studied, there is a lack of comprehensive research on the long-term impact of chronic neuroinflammation on stroke recovery and rehabilitation. Understanding how persistent inflammation affects neuroplasticity, functional recovery, and the risk of recurrent strokes is crucial. Investigating the temporal dynamics of neuroinflammation and its long-term effects could lead to better strategies for managing stroke recovery and preventing chronic neurological deficits and provide more potential therapeutic targets.

#### Chronic neuroinflammation and neuroplasticity

Research should focus on how chronic inflammation influences neuroplasticity—the brain's ability to reorganize and form new neural connections. Chronic inflammation may hinder this process, resulting in poorer recovery outcomes. Longitudinal studies and advanced imaging techniques could help elucidate these effects and identify intervention points.

#### Functional recovery

Persistent neuroinflammation might impair functional recovery by affecting motor and cognitive functions. Importantly, understanding how neuroinflammation affects the functional connections between brain regions is an active area of research and should be studied further (200). This knowledge could guide the development of therapeutic strategies to enhance recovery.

#### Recurrent stroke risk

Chronic inflammation is a risk factor for recurrent strokes (123, 201). Future research should explore how ongoing inflammatory processes contribute to this risk and identify biomarkers that can predict recurrence. Preventative strategies could then be developed to mitigate this risk in stroke survivors.

### Emerging trends in imaging neuroinflammation

Although several imaging techniques have been developed to visualize and quantify neuroinflammation, there is a need for more sensitive and specific biomarkers that can accurately reflect the extent and nature of neuroinflammation in stroke patients. Current imaging modalities, such as MRI and PET, have limitations in detecting subtle inflammatory changes and differentiating between various types of immune cell activation. Advancing imaging technology to include more precise and reliable biomarkers would improve the diagnosis, monitoring, and treatment of neuroinflammation in stroke patients.

Enhanced Sensitivity and Specificity: Developing new imaging agents and techniques that can more precisely target and visualize specific inflammatory cells and molecules is crucial. For instance, using novel PET tracers that bind to unique markers of microglial activation or specific cytokines could improve sensitivity and specificity.

#### Multimodal imaging approaches

Combining multiple imaging modalities (e.g., PET/MRI) can provide a more comprehensive picture of neuroinflammation. Research focused on integrating these technologies to leverage their strengths and may provide a more detailed and accurate assessments.

#### Standardized protocols and quantification

Establishing standardized imaging protocols and quantitative measures for neuroinflammation is essential for consistency and comparability across studies. This would facilitate the clinical application of these techniques and enhance their reliability in monitoring treatment responses.

### Identification of circulating biomarkers

The quest for a reliable biomarker of stroke has been challenging due to several factors inherent to the condition's complexity and the biological processes involved. Despite extensive research, a universally accepted, highly specific, and sensitive biomarker for stroke remains elusive. There are several currently under investigation, several of which could also provide information about the post stroke inflammatory cascade.

Glial fibrillary acidic protein (GFAP), an astrocyte-specific protein, shows promise in distinguishing between ischemic and hemorrhagic strokes (202). Elevated blood levels of GFAP are typically associated with hemorrhagic events, aiding in critical treatment decisions. Matrix metalloproteinase-9 (MMP-9) is being studied as a marker of BBB disruption and hemorrhagic transformation risk, particularly in patients treated with tissue plasminogen activator (tPA) (203, 204).

Neurofilament light chain (NfL), a structural protein found in neurons, serves as an indicator of axonal damage. Its levels in blood or cerebrospinal fluid correlate with infarct volumes and functional outcomes, making it valuable for prognostication (205). MicroRNAs, particularly miR-124 and miR-223, have also shown potential in stroke diagnosis and prognosis due to their rapid expression changes in response to cerebral ischemia (206).

While these biomarkers are promising, stroke's complexity means that no single marker is likely to provide all necessary information. Current research focuses on developing panels of multiple biomarkers and integrating this data with clinical information and advanced imaging techniques. This comprehensive approach aims to improve stroke diagnosis, prognosis, and treatment strategies, ultimately enhancing patient care and outcomes (207, 208).

### Correlate animal models with human immune state

Due to multiple technical and logistical challenges in studying the immune response from stroke patients, much of what is known about the immune response comes from animal studies. There is a paucity of human data which may explain why several promising therapies in animal models have not shown successful results in early clinical trials.

#### Cross-species comparison

Conduct systematic comparisons between the immune responses observed in animal models and those in humans. This could involve parallel studies where the same inflammatory markers and pathways are investigated in both species, using similar methodologies.

#### Translational biomarkers

Identify biomarkers that are conserved across species and can be reliably measured in both animal models and human patients. These biomarkers could serve as bridges for translating findings from preclinical to clinical settings.

#### Humanized animal models

Develop and utilize humanized animal models that better mimic the human immune system as there is mounting evidence that the human and rodent immune responses are quite divergent (209, 210). These models could provide more relevant insights into human neuroinflammatory processes and improve the predictive value of preclinical studies.

### Ongoing translational trials

The Table 1 summarizes the current ongoing clinical trials that are designed to test various hypothesis about the role of the immune system in acute stroke and that have been registered on ClinicalTrials.Gov.

**Table 1:** Selected ongoing clinical trials with current status and phase for trials related to neuroinflammation and acute cerebral ischemia.

NCT number	Study title	Summary//Status	Location and phase
NCT05277129	The Immune Response to Stroke	Observational study analyzing B and T Cell response after stroke and correlating with clinical outcome. // Recruiting	Akershus University Hospital, Lørenskog, Norway
NCT04852445	The Temporal Cellular Landscape of the Adaptive Immune System in Patients With Acute Stroke	Case Control Study seeking to determine by peripheral blood samples if patients that experience an increase in anti inflammatory Treg cells (CD4+, CD25, +FoxP3+, “brain” Treg) after stroke have improved clinical outcomes. // Recruiting.	Academisch Medisch Centrum—Universiteit van Amsterdam, Netherlands
NCT06248242	Treatment of Acute Ischemic Stroke With Rt-PA Combined With Edaravone Dexborneol	RCT prospective cohort study to assess effect of a free radical scavanger (Edaravone) and inflammatory cytokine inhibitor (Dexborneol) on outcomes post stroke. // Recruiting	The Second Hospital of Hebei Medical University, Shijiazhuang, Hebei, China
NCT03662750	Inflammatory faCtors AfteR acUte Ischemic Stroke (ICARUS)	Single-centre hospital-based cohort using serial TSPO-PET imaging, immune cell profiling to assess the degree of microglial activation and coorelate that with circulating immune bio markers. // Recruiting	Imperial College London; Institute for Stroke and Dementia Research, Munich, Germany
NCT04675762	Combination Fingolimod With Alteplase Bridging With Thrombectomy in Acute Ischemic Stroke	RCT assess the efficacy of an Immune Modulator Fingolimod (sphingosine-1-phosphate receptor modulator) is a beneficial additional treatment to alteplase and thrombectomy. // Recruiting	Zhejiang University; Hangzhou, China
NCT04321512	Study of Circulating Monocytes in Patients With Ischemic Vascular Disease	Prospective observational study to determine the levels of P2X4 monocytes, Flt-1/VEGFR-1 and CD13 monocytes, thought to be linked to post ischemic immune meditated injury, in stroke and heart attack patients. // Recruiting	University of Connecticut, CT, USA
NCT05880524	Reduction of SystemiC Inflammation After Ischemic Stroke by Intravenous DNase Administration (ReSCInD) (ReSCInD)	Randomized, placebo-controlled single-blinded, single-center trial test the hypothesis that DNase 1 administration leads to a reduction in systemic immune response measured in patients after acute ischemic stroke compared to control treatment. // Not yet recruiting.	University of Munich, Germany Phase 2

NCT, national clinical trials registry number; RCT, randomized control trial.

## Conclusion

In summary, the role of neuroinflammation in stroke pathology is multifaceted and critical to understanding stroke outcomes and potential treatments. Neuroinflammation begins immediately after the onset of a stroke, involving both the innate and adaptive immune systems. Acute neuroinflammatory responses can exacerbate brain damage, while chronic inflammation can impede recovery. Thromboinflammation, driven by platelet activation and immune cell interactions, further complicates the ischemic environment.

Diverse bodies of evidence from multiple sources implicate the post-stroke immune response as a key mediator of neurologic injury after the initial infarct. The dozens of cell types, cytokines, and messenger molecules present a myriad of potential targets for future therapies. They also present excellent options that could serve as biomarkers of neuroinflammation, and possibly prognosis, in future clinical studies.

Understanding the underlying mechanisms of neuroinflammation is key to developing these potential future therapeutics. From the moment of thrombus formation, an intricate thromboinflammatory cascade begins to recruit systemic immune cells to the site of injury. As the ischemia progresses, signaling molecules activate resident CNS immune cells (microglia) and allow for T cell and macrophage infiltration through the BBB which ultimately leads to more neuronal damage. Lastly, the potential development of autoantibodies against neural tissue or a maladaptive chronic inflammation can perpetuate an inflammatory response that eventually will fade as time progresses post injury.

The breakdown of the BBB during stroke allows peripheral immune cells and harmful substances to infiltrate the brain, contributing to edema, oxidative stress, and secondary neuronal injury. This disruption also increases the risk of infections, which are associated with poorer outcomes and higher mortality rates. Imaging techniques like PET and MRI are advancing our ability to monitor neuroinflammatory processes *in vivo*, potentially guiding personalized treatment strategies.

Current therapeutic approaches, including anti-inflammatory drugs and immunomodulatory therapies, aim to balance the need to control harmful inflammation while promoting repair and recovery. Despite promising preclinical results, translating these therapies to clinical practice remains challenging, emphasizing the need for further research and well-designed clinical trials.

Advancing imaging technology to include more precise and reliable biomarkers would improve the diagnosis, monitoring, and treatment of neuroinflammation in stroke patients. Enhanced sensitivity and specificity in imaging techniques, coupled with standardized protocols and multimodal approaches, are essential steps forward.

While much work is being done already to understand the acute phase of neuroinflammation, future research should focus on the long-term impact of neuroinflammation on stroke recovery, the development of more sensitive and specific imaging biomarkers, and improving the correlation between animal models and human immune responses. Investigating the temporal dynamics of neuroinflammation and its long-term effects could lead to better strategies for managing stroke recovery and preventing chronic neurological deficits.

Furthermore, bridging the gap between animal models and human studies is crucial. Translating promising animal studies into patients has presented numerous challenges, however, most likely because the immune mechanisms in rodents and humans are not as congruent as assumed. Systematic comparisons, identification of translational biomarkers, and the development of humanized animal models can enhance the relevance of preclinical findings and their applicability to human clinical trials.

In conclusion, addressing these challenges and opportunities will pave the way for more effective therapeutic strategies and improved outcomes for stroke patients. Through a deeper understanding of neuroinflammatory mechanisms, we can significantly impact the future of stroke treatment and recovery.

## References

[1] Renedo D Acosta JN Leasure AC Sharma R Krumholz HM de Havenon A Burden of ischemic and hemorrhagic stroke across the US from 1990 to 2019. JAMA Neurol. (2024) 81:394–404. 10.1001/jamaneurol.2024.0190 38436973 PMC10913004

[2] Fang MC Chang Y Hylek EM Rosand J Greenberg SM Go AS Advanced age, anticoagulation intensity, and risk for intracranial hemorrhage among patients taking warfarin for atrial fibrillation. Ann Intern Med. (2004) 141:745–52. 10.7326/0003-4819-141-10-200411160-00005 15545674

[3] Virani SS Alonso A Benjamin EJ Bittencourt MS Callaway CW Carson AP Heart disease and stroke statistics—2020 update: a report from the American heart association. Circulation. (2020) 141:e139–596. 10.1161/CIR.0000000000000757 31992061

[4] Andersen KK Olsen TS Dehlendorff C Kammersgaard LP. Hemorrhagic and ischemic strokes compared. Stroke. (2009) 40:2068–72. 10.1161/STROKEAHA.108.540112 19359645

[5] Feigin VL Krishnamurthi R. Stroke is largely preventable across the globe: where to next? Lancet. (2016) 388:733–4. 10.1016/S0140-6736(16)30679-1 27431357

[6] Johnson CO Nguyen M Roth GA Nichols E Alam T Abate D Global, regional, and national burden of stroke, 1990–2016: a systematic analysis for the global burden of disease study 2016. Lancet Neurol. (2019) 18:439–58. 10.1016/S1474-4422(19)30034-1 30871944 PMC6494974

[7] Lucas-Noll J Clua-Espuny JL Lleixà-Fortuño M Gavaldà-Espelta E Queralt-Tomas L Panisello-Tafalla A The costs associated with stroke care continuum: a systematic review. Health Econ Rev. (2023) 13:32. 10.1186/s13561-023-00439-6 37193926 PMC10190015

[8] Rochmah TN Rahmawati IT Dahlui M Budiarto W Bilqis N. Economic burden of stroke disease: a systematic review. Int J Environ Res Public Health. (2021) 18:7552. 10.3390/ijerph18147552 34299999 PMC8307880

[9] Kauw F Heit JJ Martin BW van Ommen F Kappelle LJ Velthuis BK Computed tomography perfusion data for acute ischemic stroke evaluation using rapid software: pitfalls of automated postprocessing. J Comput Assist Tomogr. (2020) 44:75–7. 10.1097/RCT.0000000000000946 31804241

[10] Sander L Pezold S Andermatt S Amann M Meier D Wendebourg MJ Accurate, rapid and reliable, fully automated MRI brainstem segmentation for application in multiple sclerosis and neurodegenerative diseases. Hum Brain Mapp. (2019) 40:4091–104. 10.1002/hbm.24687 31206931 PMC6865769

[11] Albers GW Marks MP Kemp S Christensen S Tsai JP Ortega-Gutierrez S Thrombectomy for stroke at 6 to 16 hours with selection by perfusion imaging. N Engl J Med. (2018) 378:708–18. 10.1056/NEJMoa1713973 29364767 PMC6590673

[12] Fayad P. Improved prospects for thrombectomy in large ischemic stroke. N Engl J Med. (2023) 388:1326–8. 10.1056/NEJMe2300193 36762847

[13] Heit JJ Mlynash M Christensen S Kemp SM Lansberg MG Marks MP What predicts poor outcome after successful thrombectomy in late time windows? J Neurointerv Surg. (2021) 13:421–5. 10.1136/neurintsurg-2020-016125 32554693

[14] Moskowitz MA Lo EH Iadecola C. The science of stroke: mechanisms in search of treatments. Neuron. (2010) 67:181–98. 10.1016/j.neuron.2010.07.002 20670828 PMC2957363

[15] Lo EH. Experimental models, neurovascular mechanisms and translational issues in stroke research. Br J Pharmacol. (2008) 153(Suppl 1):S396–405. 10.1038/sj.bjp.0707626 18157168 PMC2268069

[16] Stonesifer C Corey S Ghanekar S Diamandis Z Acosta SA Borlongan CV. Stem cell therapy for abrogating stroke-induced neuroinflammation and relevant secondary cell death mechanisms. Prog Neurobiol. (2017) 158:94–131. 10.1016/j.pneurobio.2017.07.004 28743464 PMC5671910

[17] Zhao S Li F Leak RK Chen J Hu X. Regulation of neuroinflammation through programed death-1/programed death ligand signaling in neurological disorders. Front Cell Neurosci. (2014) 8:271. 10.3389/fncel.2014.00271 25232304 PMC4153295

[18] Prakash R Carmichael ST. Blood–brain barrier breakdown and neovascularization processes after stroke and traumatic brain injury. Curr Opin Neurol. (2015) 28:556–64. 10.1097/WCO.0000000000000248 26402408 PMC5267616

[19] Liang Z Lou Y Hao Y Li H Feng J Liu S. The relationship of astrocytes and microglia with different stages of ischemic stroke. Curr Neuropharmacol. (2023) 21:2465–80. 10.2174/1570159X21666230718104634 37464832 PMC10616922

[20] Jia J Yang L Chen Y Zheng L Chen Y Xu Y The role of microglial phagocytosis in ischemic stroke. Front Immunol. (2022) 12:790201. 10.3389/fimmu.2021.790201 35082781 PMC8784388

[21] Li X Chen G. CNS-peripheral immune interactions in hemorrhagic stroke. J Cereb Blood Flow Metab. (2023) 43:185–97. 10.1177/0271678X221145089 36476130 PMC9903219

[22] Toubai T Mathewson ND Magenau J Reddy P. Danger signals and graft-versus-host disease: current understanding and future perspectives. Front Immunol. (2016) 7:539. 10.3389/fimmu.2016.00539 27965667 PMC5126092

[23] Bu Z-Q Yu H-Y Wang J He X Cui Y-R Feng J-C Emerging role of ferroptosis in the pathogenesis of ischemic stroke: a new therapeutic target? ASN Neuro. (2021) 13:17590914211037505. 10.1177/17590914211037505 34463559 PMC8424725

[24] Hu X Bao Y Li M Zhang W Chen C. The role of ferroptosis and its mechanism in ischemic stroke. Exp Neurol. (2024) 372:114630. 10.1016/j.expneurol.2023.114630 38056585

[25] Liu J Guo Z-N Yan X-L Huang S Ren J-X Luo Y Crosstalk between autophagy and ferroptosis and its putative role in ischemic stroke. Front Cell Neurosci. (2020) 14:577403. 10.3389/fncel.2020.577403 33132849 PMC7566169

[26] Dostovic Z Dostovic E Smajlovic D Ibrahimagic OC Avdic L. Brain edema after ischaemic stroke. Med Arch. (2016) 70:339–41. 10.5455/medarh.2016.70.339-341 27994292 PMC5136437

[27] Yao Y Zhang Y Liao X Yang R Lei Y Luo J. Potential therapies for cerebral edema after ischemic stroke: a mini review. Front Aging Neurosci. (2021) 12:618819. 10.3389/fnagi.2020.618819 33613264 PMC7890111

[28] Schuhmann MK Guthmann J Stoll G Nieswandt B Kraft P Kleinschnitz C. Blocking of platelet glycoprotein receptor Ib reduces “thrombo-inflammation” in mice with acute ischemic stroke. J Neuroinflammation. (2017) 14:18. 10.1186/s12974-017-0792-y 28109273 PMC5251224

[29] Steubing RD Szepanowski F David C Mohamud Yusuf A Mencl S Mausberg A-K Platelet depletion does not alter long-term functional outcome after cerebral ischaemia in mice. Brain Behav Immun Health. (2022) 24:100493. 10.1016/j.bbih.2022.100493 35928516 PMC9343933

[30] Planas AM. Role of immune cells migrating to the ischemic brain. Stroke. (2018) 49:2261–7. 10.1161/STROKEAHA.118.021474 30355002

[31] Szepanowski RD Haupeltshofer S Vonhof SE Frank B Kleinschnitz C Casas AI. Thromboinflammatory challenges in stroke pathophysiology. Semin Immunopathol. (2023) 45:389–410. 10.1007/s00281-023-00994-4 37273022 PMC10241149

[32] Massberg S Grahl L von Bruehl M-L Manukyan D Pfeiler S Goosmann C Reciprocal coupling of coagulation and innate immunity via neutrophil serine proteases. Nat Med. (2010) 16:887–96. 10.1038/nm.2184 20676107

[33] von Brühl M-L Stark K Steinhart A Chandraratne S Konrad I Lorenz M Monocytes, neutrophils, and platelets cooperate to initiate and propagate venous thrombosis in mice in vivo. J Exp Med. (2012) 209:819–35. 10.1084/jem.20112322 22451716 PMC3328366

[34] Vindegaard N Muñoz-Briones C El Ali HH Kristensen LK Rasmussen RS Johansen FF T-cells and macrophages peak weeks after experimental stroke: spatial and temporal characteristics. Neuropathology. (2017) 37:407–14. 10.1111/neup.12387 28517732

[35] Shichita T Sugiyama Y Ooboshi H Sugimori H Nakagawa R Takada I Pivotal role of cerebral interleukin-17-producing gammadeltaT cells in the delayed phase of ischemic brain injury. Nat Med. (2009) 15:946–50. 10.1038/nm.1999 19648929

[36] Gelderblom M Weymar A Bernreuther C Velden J Arunachalam P Steinbach K Neutralization of the IL-17 axis diminishes neutrophil invasion and protects from ischemic stroke. Blood. (2012) 120:3793–802. 10.1182/blood-2012-02-412726 22976954

[37] Xie L Li W Hersh J Liu R Yang S-H. Experimental ischemic stroke induces long-term T cell activation in the brain. J Cereb Blood Flow Metab. (2019) 39:2268–76. 10.1177/0271678X18792372 30092705 PMC6827125

[38] Selvaraj UM Poinsatte K Torres V Ortega SB Stowe AM. Heterogeneity of B cell functions in stroke-related risk, prevention, injury, and repair. Neurotherapeutics. (2016) 13:729–47. 10.1007/s13311-016-0460-4 27492770 PMC5081124

[39] Fauchais A-L Lalloué F Lise M-C Boumediene A Preud’homme J-L Vidal E Role of endogenous brain-derived neurotrophic factor and sortilin in B cell survival. J Immunol. (2008) 181:3027–38. 10.4049/jimmunol.181.5.3027 18713973

[40] Kronenberg G Uhlemann R Richter N Klempin F Wegner S Staerck L Distinguishing features of microglia- and monocyte-derived macrophages after stroke. Acta Neuropathol. (2018) 135:551–68. 10.1007/s00401-017-1795-6 29249001

[41] Li L-Z Huang Y-Y Yang Z-H Zhang S-J Han Z-P Luo Y-M. Potential microglia-based interventions for stroke. CNS Neurosci Ther. (2020) 26:288–96. 10.1111/cns.13291 32064759 PMC7052807

[42] Werner Y Mass E Ashok Kumar P Ulas T Händler K Horne A Cxcr4 distinguishes HSC-derived monocytes from microglia and reveals monocyte immune responses to experimental stroke. Nat Neurosci. (2020) 23:351–62. 10.1038/s41593-020-0585-y 32042176 PMC7523735

[43] Berchtold D Priller J Meisel C Meisel A. Interaction of microglia with infiltrating immune cells in the different phases of stroke. Brain Pathol. (2020) 30:1208–18. 10.1111/bpa.12911 33058417 PMC8018083

[44] Živančević K Lović D Andjus PR Radenović L. Neuroinflammation in post-ischemic brain. In: Pluta R, editor. Cerebral Ischemia. Brisbane (AU): Exon Publications (2021). 34905311

[45] Candelario-Jalil E Dijkhuizen RM Magnus T. Neuroinflammation, stroke, blood-brain barrier dysfunction, and imaging modalities. Stroke. (2022) 53:1473–86. 10.1161/STROKEAHA.122.036946 35387495 PMC9038693

[46] Akamatsu Y Chaitin HJ Hanafy KA. Post-stroke recrudescence-a possible connection to autoimmunity? Rev Neurosci. (2022) 33:207–12. 10.1515/revneuro-2021-0062 34363383

[47] Sobowale OA Parry-Jones AR Smith CJ Tyrrell PJ Rothwell NJ Allan SM. Interleukin-1 in stroke. Stroke. (2016) 47:2160–7. 10.1161/STROKEAHA.115.010001 26931154

[48] Anthony S Cabantan D Monsour M Borlongan CV. Neuroinflammation, stem cells, and stroke. Stroke. (2022) 53:1460–72. 10.1161/STROKEAHA.121.036948 35380050 PMC9038685

[49] Vargas M Horcajada JP Obach V Revilla M Cervera A Torres F Clinical consequences of infection in patients with acute stroke: is it prime time for further antibiotic trials? Stroke. (2006) 37:461–5. 10.1161/01.STR.0000199138.73365.b3 16385093

[50] Vermeij J-D Westendorp WF Dippel DW van de Beek D Nederkoorn PJ. Antibiotic therapy for preventing infections in people with acute stroke. Cochrane Database Syst Rev. (2018) 1:CD008530. 10.1002/14651858.CD008530.pub3 29355906 PMC6491314

[51] Westendorp WF Dames C Nederkoorn PJ Meisel A. Immunodepression, infections, and functional outcome in ischemic stroke. Stroke. (2022) 53:1438–48. 10.1161/STROKEAHA.122.038867 35341322

[52] De Meyer SF Langhauser F Haupeltshofer S Kleinschnitz C Casas AI. Thromboinflammation in brain ischemia: recent updates and future perspectives. Stroke. (2022) 53:1487–99. 10.1161/STROKEAHA.122.038733 35360931

[53] Kleinschnitz C De Meyer SF Schwarz T Austinat M Vanhoorelbeke K Nieswandt B Deficiency of von Willebrand factor protects mice from ischemic stroke. Blood. (2009) 113:3600–3. 10.1182/blood-2008-09-180695 19182208

[54] Zhao B-Q Chauhan AK Canault M Patten IS Yang JJ Dockal M von Willebrand factor-cleaving protease ADAMTS13 reduces ischemic brain injury in experimental stroke. Blood. (2009) 114:3329–34. 10.1182/blood-2009-03-213264 19687510 PMC2759655

[55] Kleinschnitz C Pozgajova M Pham M Bendszus M Nieswandt B Stoll G. Targeting platelets in acute experimental stroke: impact of glycoprotein Ib, VI, and IIb/IIIa blockade on infarct size, functional outcome, and intracranial bleeding. Circulation. (2007) 115:2323–30. 10.1161/CIRCULATIONAHA.107.691279 17438148

[56] Denorme F Martinod K Vandenbulcke A Denis CV Lenting PJ Deckmyn H The von Willebrand factor A1 domain mediates thromboinflammation, aggravating ischemic stroke outcome in mice. Haematologica. (2020) 106:819–28. 10.3324/haematol.2019.241042 PMC7927893 32107335

[57] Li T-T Fan M-L Hou S-X Li X-Y Barry DM Jin H A novel snake venom-derived GPIb antagonist, anfibatide, protects mice from acute experimental ischaemic stroke and reperfusion injury. Br J Pharmacol. (2015) 172:3904–16. 10.1111/bph.13178 25917571 PMC4523344

[58] Alshehri OM Hughes CE Montague S Watson SK Frampton J Bender M Fibrin activates GPVI in human and mouse platelets. Blood. (2015) 126:1601–8. 10.1182/blood-2015-04-641654 26282541 PMC4582337

[59] Shattil SJ Kim C Ginsberg MH. The final steps of integrin activation: the end game. Nat Rev Mol Cell Biol. (2010) 11:288–300. 10.1038/nrm2871 20308986 PMC3929966

[60] Offermanns S. Activation of platelet function through G protein–coupled receptors. Circ Res. (2006) 99:1293–304. 10.1161/01.RES.0000251742.71301.16 17158345

[61] Müller F Mutch NJ Schenk WA Smith SA Esterl L Spronk HM Platelet polyphosphates are proinflammatory and procoagulant mediators in vivo. Cell. (2009) 139:1143–56. 10.1016/j.cell.2009.11.001 20005807 PMC2796262

[62] Rolfes V Ribeiro LS Hawwari I Böttcher L Rosero N Maasewerd S Platelets fuel the inflammasome activation of innate immune cells. Cell Rep. (2020) 31:107615. 10.1016/j.celrep.2020.107615 32402278 PMC7225754

[63] Lindemann S Tolley ND Dixon DA McIntyre TM Prescott SM Zimmerman GA Activated platelets mediate inflammatory signaling by regulated interleukin 1β synthesis. J Cell Biol. (2001) 154:485–90. 10.1083/jcb.200105058 11489912 PMC2196422

[64] Said S Rosenblum WI Povlishock JT Nelson GH. Correlations between morphological changes in platelet aggregates and underlying endothelial damage in cerebral microcirculation of mice. Stroke. (1993) 24:1968–76. 10.1161/01.str.24.12.1968 8248979

[65] Braun LJ Stegmeyer RI Schäfer K Volkery S Currie SM Kempe B Platelets docking to VWF prevent leaks during leukocyte extravasation by stimulating Tie-2. Blood. (2020) 136:627–39. 10.1182/blood.2019003442 32369573 PMC7413753

[66] Golias C Charalabopoulos A Stagikas D Charalabopoulos K Batistatou A. The kinin system–bradykinin: biological effects and clinical implications. Multiple role of the kinin system–bradykinin. Hippokratia. (2007) 11:124–8. 19582206 PMC2658795

[67] Alexander-Curtis M Pauls R Chao J Volpi JJ Bath PM Verdoorn TA. Human tissue kallikrein in the treatment of acute ischemic stroke. Ther Adv Neurol Disord. (2019) 12:1756286418821918. 10.1177/1756286418821918 30719079 PMC6348491

[68] Simão F Feener EP. The effects of the contact activation system on hemorrhage. Front Med. (2017) 4:121. 10.3389/fmed.2017.00121 PMC5534673 28824910

[69] Nokkari A Abou-El-Hassan H Mechref Y Mondello S Kindy MS Jaffa AA Implication of the kallikrein-kinin system in neurological disorders: quest for potential biomarkers and mechanisms. Prog Neurobiol. (2018) 165–167:26–50. 10.1016/j.pneurobio.2018.01.003 29355711 PMC6026079

[70] DiaMedica therapeutics announces first patient dosed in relaunch of its pivotal phase 2/3 ReMEDy2 Trial of DM199 for the treatment of acute ischemic stroke. DiaMedica Therapeutics, Inc. (2024). Available online at: https://www.diamedica.com/investors/press-releases/detail/1678/diamedica-therapeutics-announces-first-patient-dosed-in (accessed July 30, 2024).

[71] ReMEDy1 NCT03290560 evaluation to assess safety and tolerability of DM199 in subjects with acute ischemic stroke | ClinicalTrials.gov. Available online at: https://clinicaltrials.gov/study/NCT03290560 (accessed July 30, 2024).

[72] ReMEDy2 Trial, NCT05065216 | ClinicalTrials.gov. Available online at: https://clinicaltrials.gov/study/NCT05065216?tab=results (accessed July 30, 2024).

[73] Mracsko E Liesz A Stojanovic A Lou WP-K Osswald M Zhou W Antigen dependently activated cluster of differentiation 8-positive T cells cause perforin-mediated neurotoxicity in experimental stroke. J Neurosci. (2014) 34:16784–95. 10.1523/JNEUROSCI.1867-14.2014 25505331 PMC6608504

[74] Liesz A Zhou W Mracskó É Karcher S Bauer H Schwarting S Inhibition of lymphocyte trafficking shields the brain against deleterious neuroinflammation after stroke. Brain. (2011) 134:704–20. 10.1093/brain/awr008 21354973

[75] Walter J Mende J Hutagalung S Alhalabi OT Grutza M Zheng G The single-dose application of interleukin-4 ameliorates secondary brain damage in the early phase after moderate experimental traumatic brain injury in mice. Int J Mol Sci. (2023) 24:12756. 10.3390/ijms241612756 37628939 PMC10454634

[76] Li P Mao L Liu X Gan Y Zheng J Thomson AW Essential role of program death 1-ligand 1 in regulatory T-cell–afforded protection against blood–brain barrier damage after stroke. Stroke. (2014) 45:857–64. 10.1161/STROKEAHA.113.004100 24496394 PMC3939692

[77] Burda JE Sofroniew MV. Reactive gliosis and the multicellular response to CNS damage and disease. Neuron. (2014) 81:229–48. 10.1016/j.neuron.2013.12.034 24462092 PMC3984950

[78] Walsh JT Hendrix S Boato F Smirnov I Zheng J Lukens JR MHCII-independent CD4+ T cells protect injured CNS neurons via IL-4. J Clin Invest. (2015) 125:699–714. 10.1172/JCI76210 25607842 PMC4319416

[79] Filiano AJ Xu Y Tustison NJ Marsh RL Baker W Smirnov I Unexpected role of interferon-γ in regulating neuronal connectivity and social behaviour. Nature. (2016) 535:425–9. 10.1038/nature18626 27409813 PMC4961620

[80] Engler-Chiurazzi EB Monaghan KL Wan ECK Ren X. Role of B cells and the aging brain in stroke recovery and treatment. GeroScience. (2020) 42:1199–216. 10.1007/s11357-020-00242-9 32767220 PMC7525651

[81] Ren X Akiyoshi K Vandenbark AA Hurn PD Offner H. Regulatory B cells limit CNS inflammation and neurologic deficits in murine experimental stroke. J Neurosci. (2011) 31:8556–63. 10.1523/JNEUROSCI.1623-11.2011 21653859 PMC3111929

[82] Woods D Jiang Q Chu X-P. Monoclonal antibody as an emerging therapy for acute ischemic stroke. Int J Physiol Pathophysiol Pharmacol. (2020) 12:95–106. 32934765 PMC7486556

[83] Elkind MSV Veltkamp R Montaner J Johnston SC Singhal AB Becker K Natalizumab in acute ischemic stroke (ACTION II): a randomized, placebo-controlled trial. Neurology. (2020) 95:e1091–104. 10.1212/WNL.0000000000010038 32591475 PMC7668547

[84] Llovera G Hofmann K Roth S Salas-Pérdomo A Ferrer-Ferrer M Perego C Results of a preclinical randomized controlled multicenter trial (pRCT): anti-CD49d treatment for acute brain ischemia. Sci Transl Med. (2015) 7. 10.1126/scitranslmed.aaa9853 26246166

[85] del Zoppo GJ. Lessons from stroke trials using anti-inflammatory approaches that have failed. Ernst Schering Res Found Workshop. (2004) 47:155–84. 10.1007/978-3-662-05426-0_9 15032059

[86] Gill D Veltkamp R. Dynamics of T cell responses after stroke. Curr Opin Pharmacol. (2016) 26:26–32. 10.1016/j.coph.2015.09.009 26452204

[87] Zhang D Ren J Luo Y He Q Zhao R Chang J T cell response in ischemic stroke: from mechanisms to translational insights. Front Immunol. (2021) 12:707972. 10.3389/fimmu.2021.707972 34335623 PMC8320432

[88] Kim E Cho S. CNS and peripheral immunity in cerebral ischemia: partition and interaction. Exp Neurol. (2021) 335:113508. 10.1016/j.expneurol.2020.113508 33065078 PMC7750306

[89] Yu H Cai Y Zhong A Zhang Y Zhang J Xu S. The “dialogue” between central and peripheral immunity after ischemic stroke: focus on spleen. Front Immunol. (2021) 12:792522. 10.3389/fimmu.2021.792522 34975893 PMC8717871

[90] Kim E Yang J Beltran CD Cho S. Role of spleen-derived monocytes/macrophages in acute ischemic brain injury. J Cereb Blood Flow Metab. (2014) 34:1411–9. 10.1038/jcbfm.2014.101 24865998 PMC4126087

[91] Nian K Harding IC Herman IM Ebong EE. Blood-brain barrier damage in ischemic stroke and its regulation by endothelial mechanotransduction. Front Physiol. (2020) 11:605398. 10.3389/fphys.2020.605398 33424628 PMC7793645

[92] Fukuma K Yamagami H Ihara M Tanaka T Miyata T Miyata S P2y12 reaction units and clinical outcomes in acute large artery atherosclerotic stroke: a multicenter prospective study. J Atheroscler Thromb. (2023) 30:39–55. 10.5551/jat.63369 35249906 PMC9899699

[93] Li Z-X Xiong Y Gu H-Q Fisher M Xian Y Johnston SC P2y12 inhibitors plus aspirin versus aspirin alone in patients with minor stroke or high-risk transient ischemic attack. Stroke. (2021) 52:2250–7. 10.1161/STROKEAHA.120.033040 34039032

[94] Wichaiyo S Parichatikanond W Rattanavipanon W. Glenzocimab: a GPVI (glycoprotein VI)-targeted potential antiplatelet agent for the treatment of acute ischemic stroke. Stroke. (2022) 53:3506–13. 10.1161/STROKEAHA.122.039790 36128904

[95] Fu Y Hao J Zhang N Ren L Sun N Li Y-J Fingolimod for the treatment of intracerebral hemorrhage: a 2-arm proof-of-concept study. JAMA Neurol. (2014) 71:1092–101. 10.1001/jamaneurol.2014.1065 25003359

[96] Wei Y Yemisci M Kim H-H Yung LM Shin HK Hwang S-K Fingolimod provides long-term protection in rodent models of cerebral ischemia. Ann Neurol. (2011) 69:119–29. 10.1002/ana.22186 21280082 PMC3200194

[97] Davoine C Bouckaert C Fillet M Pochet L. Factor XII/XIIa inhibitors: their discovery, development, and potential indications. Eur J Med Chem. (2020) 208:112753. 10.1016/j.ejmech.2020.112753 32883641

[98] Craig TJ Levy DS Reshef A Lumry WR Martinez-Saguer I Jacobs JS Garadacimab for hereditary angioedema attack prevention: long-term efficacy, quality of life, and safety data from a phase 2, randomised, open-label extension study. Lancet Haematol. (2024) 11:e436–47. 10.1016/S2352-3026(24)00081-4 38710185

[99] Papi A Stapleton RD Shore PM Bica MA Chen Y Larbig M Efficacy and safety of garadacimab in combination with standard of care treatment in patients with severe COVID-19. Lung. (2023) 201:159–70. 10.1007/s00408-023-00615-9 37000214 PMC10064633

[100] Marcos-Contreras OA Martinez de Lizarrondo S Bardou I Orset C Pruvost M Anfray A Hyperfibrinolysis increases blood–brain barrier permeability by a plasmin- and bradykinin-dependent mechanism. Blood. (2016) 128:2423–34. 10.1182/blood-2016-03-705384 27531677

[101] García-Culebras A Durán-Laforet V Peña-Martínez C Moraga A Ballesteros I Cuartero MI Role of TLR4 (toll-like receptor 4) in N1/N2 neutrophil programming after stroke. Stroke. (2019) 50:2922–32. 10.1161/STROKEAHA.119.025085 31451099

[102] Hernández-Jiménez M Abad-Santos F Cotgreave I Gallego J Jilma B Flores A Safety and efficacy of ApTOLL in patients with ischemic stroke undergoing endovascular treatment: a phase 1/2 randomized clinical trial. JAMA Neurol. (2023) 80:779–88. 10.1001/jamaneurol.2023.1660 37338893 PMC10282959

[103] Ridker PM Everett BM Thuren T MacFadyen JG Chang WH Ballantyne C Antiinflammatory therapy with canakinumab for atherosclerotic disease. N Engl J Med. (2017) 377:1119–31. 10.1056/NEJMoa1707914 28845751

[104] Bai P Zhu R Wang P Jiang F Zhen J Yao Y The efficacy and safety of fingolimod plus standardized treatment versus standardized treatment alone for acute ischemic stroke: a systematic review and meta-analysis. Pharmacol Res Perspect. (2022) 10:e00972. 10.1002/prp2.972 35585652 PMC9117458

[105] Marcet P Santos N Borlongan CV. When friend turns foe: central and peripheral neuroinflammation in central nervous system injury. Neuroimmunol Neuroinflamm. (2017) 4:82–92. 10.20517/2347-8659.2017.07 29670933 PMC5901724

[106] Xie M Hao Y Feng L Wang T Yao M Li H Neutrophil heterogeneity and its roles in the inflammatory network after ischemic stroke. Curr Neuropharmacol. (2023) 21:621–50. 10.2174/1570159X20666220706115957 35794770 PMC10207908

[107] Poh XY Loh FK Friedland JS Ong CWM. Neutrophil-mediated immunopathology and matrix metalloproteinases in central nervous system – tuberculosis. Front Immunol. (2022) 12:788976. 10.3389/fimmu.2021.788976 35095865 PMC8789671

[108] Rosell A Cuadrado E Ortega-Aznar A Hernández-Guillamon M Lo EH Montaner J. MMP-9-positive neutrophil infiltration is associated to blood-brain barrier breakdown and basal lamina type IV collagen degradation during hemorrhagic transformation after human ischemic stroke. Stroke. (2008) 39:1121–6. 10.1161/STROKEAHA.107.500868 18323498

[109] Herisson F Frodermann V Courties G Rohde D Sun Y Vandoorne K Direct vascular channels connect skull bone marrow and the brain surface enabling myeloid cell migration. Nat Neurosci. (2018) 21:1209–17. 10.1038/s41593-018-0213-2 30150661 PMC6148759

[110] Beschorner R Schluesener HJ Gözalan F Meyermann R Schwab JM. Infiltrating CD14+ monocytes and expression of CD14 by activated parenchymal microglia/macrophages contribute to the pool of CD14+ cells in ischemic brain lesions. J Neuroimmunol. (2002) 126:107–15. 10.1016/s0165-5728(02)00046-2 12020962

[111] Lyu J Xie D Bhatia TN Leak RK Hu X Jiang X. Microglial/macrophage polarization and function in brain injury and repair after stroke. CNS Neurosci Ther. (2021) 27:515–27. 10.1111/cns.13620 33650313 PMC8025652

[112] Ma K Guo J Wang G Ni Q Liu X. Toll-like receptor 2–mediated autophagy promotes microglial cell death by modulating the microglial M1/M2 phenotype. Inflammation. (2020) 43:701–11. 10.1007/s10753-019-01152-5 31834572

[113] Beuker C Schafflick D Strecker J-K Heming M Li X Wolbert J Stroke induces disease-specific myeloid cells in the brain parenchyma and pia. Nat Commun. (2022) 13:945. 10.1038/s41467-022-28593-1 35177618 PMC8854573

[114] Jiang C-T Wu W-F Deng Y-H Ge J-W. Modulators of microglia activation and polarization in ischemic stroke. Mol Med Rep. (2020) 21:2006–18. 10.3892/mmr.2020.11003 32323760 PMC7115206

[115] Sierra A Paolicelli RC Kettenmann H. Cien Años de Microglía: milestones in a century of microglial research. Trends Neurosci. (2019) 42:778–92. 10.1016/j.tins.2019.09.004 31635851

[116] Masuda T Croom D Hida H Kirov SA. Capillary blood flow around microglial somata determines dynamics of microglial processes in ischemic conditions. Glia. (2011) 59:1744–53. 10.1002/glia.21220 21800362 PMC3174346

[117] Hammond TR Dufort C Dissing-Olesen L Giera S Young A Wysoker A Single-Cell RNA sequencing of microglia throughout the mouse lifespan and in the injured brain reveals complex cell-state changes. Immunity. (2019) 50:253–71.e6. 10.1016/j.immuni.2018.11.004 30471926 PMC6655561

[118] Goverman J. Autoimmune T cell responses in the central nervous system. Nat Rev Immunol. (2009) 9:393. 10.1038/nri2550 19444307 PMC2813731

[119] Grønberg NV Johansen FF Kristiansen U Hasseldam H. Leukocyte infiltration in experimental stroke. J Neuroinflammation. (2013) 10:892. 10.1186/1742-2094-10-115 PMC3852747 24047275

[120] Schuhmann MK Langhauser F Kraft P Kleinschnitz C. B cells do not have a major pathophysiologic role in acute ischemic stroke in mice. J Neuroinflammation. (2017) 14:112. 10.1186/s12974-017-0890-x 28576128 PMC5457733

[121] Ortega SB Noorbhai I Poinsatte K Kong X Anderson A Monson NL Stroke induces a rapid adaptive autoimmune response to novel neuronal antigens. Discov Med. (2015) 19:381–92. 26105701 PMC4692161

[122] Al Mamun A Chauhan A Qi S Ngwa C Xu Y Sharmeen R Microglial IRF5-IRF4 regulator*y* axis regulates neuroinflammation after cerebral ischemia and impacts stroke outcomes. Proc Natl Acad Sci U S A. (2020) 117:1742–52. 10.1073/pnas.1914742117 31892541 PMC6983422

[123] Stuckey SM Ong LK Collins-Praino LE Turner RJ. Neuroinflammation as a key driver of secondary neurodegeneration following stroke? Int J Mol Sci. (2021) 22:13101. 10.3390/ijms222313101 34884906 PMC8658328

[124] Holmegaard L Jensen C Pedersen A Blomstrand C Blennow K Zetterberg H Circulating levels of neurofilament light chain as a biomarker of infarct and white matter hyperintensity volumes after ischemic stroke. Sci Rep. (2024) 14:16180. 10.1038/s41598-024-67232-1 39003344 PMC11246414

[125] Selçuk Ö Yayla V Çabalar M Güzel V Uysal S Gedikbaşi A. The relationship of serum S100B levels with infarction size and clinical outcome in acute ischemic stroke patients. Noro Psikiyatr Ars. (2014) 51:395–400. 10.5152/npa.2014.7213 28360660 PMC5353176

[126] Benakis C Simats A Tritschler S Heindl S Besson-Girard S Llovera G T cells modulate the microglial response to brain ischemia. Elife. (2022) 11:e82031. 10.7554/eLife.82031 36512388 PMC9747154

[127] Zhang J Jiang Y Zhu J Wu J Meng R Ji X. T cell interactions with microglia in immune-inflammatory processes of ischemic stroke. Neural Regen Res. (2025) 20:781. 10.4103/NRR.NRR-D-23-01385 PMC11624874 39075894

[128] Sanmarco LM Polonio CM Wheeler MA Quintana FJ. Functional immune cell–astrocyte interactions. J Exp Med. (2021) 218:e20202715. 10.1084/jem.20202715 34292315 PMC8302447

[129] Hersh J Yang S-H. Glia–immune interactions post-ischemic stroke and potential therapies. Exp Biol Med. (2018) 243:1302–12. 10.1177/1535370218818172 PMC6348596 30537868

[130] Lee H-G Lee J-H Flausino LE Quintana FJ. Neuroinflammation: an astrocyte perspective. Sci Transl Med. (2023) 15:eadi7828. 10.1126/scitranslmed.adi7828 37939162

[131] Jian Z Liu R Zhu X Smerin D Zhong Y Gu L The involvement and therapy target of immune cells after ischemic stroke. Front Immunol. (2019) 10:2167. 10.3389/fimmu.2019.02167 31572378 PMC6749156

[132] Xiong Y Fu Y Li Z Zheng Y Cui M Zhang C Laquinimod inhibits microglial activation, astrogliosis, BBB damage, and infarction and improves neurological damage after ischemic stroke. ACS Chem Neurosci. (2023) 14:1992–2007. 10.1021/acschemneuro.2c00740 37161270

[133] Chen Y Cai Z Ke Z. Antineuroinflammation of minocycline in stroke. Neurologist. (2017) 22:120–6. 10.1097/NRL.0000000000000136 28644252

[134] Borlongan MC Kingsbury C Salazar FE Toledo ARL Monroy GR Sadanandan N IL-2/IL-2R antibody complex enhances Treg-induced neuroprotection by dampening TNF-α inflammation in an in vitro stroke model. Neuromolecular Med. (2021) 23:540–8. 10.1007/s12017-021-08656-0 33830475 PMC8613084

[135] Shi L Sun Z Su W Xu F Xie D Zhang Q Treg cell-derived osteopontin promotes microglia-mediated white matter repair after ischemic stroke. Immunity. (2021) 54:1527–42.e8. 10.1016/j.immuni.2021.04.022 34015256 PMC8282725

[136] Kaneko Y Lee J-Y Tajiri N Tuazon JP Lippert T Russo E Translating intracarotid artery transplantation of bone marrow-derived NCS-01 cells for ischemic stroke: behavioral and histological readouts and mechanistic insights into stem cell therapy. Stem Cells Transl Med. (2020) 9:203–20. 10.1002/sctm.19-0229 31738023 PMC6988762

[137] Abe T Aburakawa D Niizuma K Iwabuchi N Kajitani T Wakao S Intravenously transplanted human multilineage-differentiating stress-enduring cells afford brain repair in a mouse lacunar stroke model. Stroke. (2020) 51:601–11. 10.1161/STROKEAHA.119.026589 31826733

[138] Xu K Lee J-Y Kaneko Y Tuazon JP Vale F van Loveren H Human stem cells transplanted into the rat stroke brain migrate to the spleen via lymphatic and inflammation pathways. Haematologica. (2019) 104:1062–73. 10.3324/haematol.2018.206581 30514806 PMC6518907

[139] Kahles T Brandes RP. NADPH oxidases as therapeutic targets in ischemic stroke. Cell Mol Life Sci. (2012) 69:2345–63. 10.1007/s00018-012-1011-8 22618244 PMC11114534

[140] Kahles T Luedike P Endres M Galla H-J Steinmetz H Busse R NADPH oxidase plays a central role in blood-brain barrier damage in experimental stroke. Stroke. (2007) 38:3000–6. 10.1161/STROKEAHA.107.489765 17916764

[141] Montaner J Ramiro L Simats A Hernández-Guillamon M Delgado P Bustamante A Matrix metalloproteinases and ADAMs in stroke. Cell Mol Life Sci. (2019) 76:3117–40. 10.1007/s00018-019-03175-5 31165904 PMC11105215

[142] Yang C Hawkins KE Doré S Candelario-Jalil E. Neuroinflammatory mechanisms of blood-brain barrier damage in ischemic stroke. Am J Physiol Cell Physiol. (2019) 316:C135–53. 10.1152/ajpcell.00136.2018 30379577 PMC6397344

[143] Montaner J Molina CA Monasterio J Abilleira S Arenillas JF Ribó M Matrix metalloproteinase-9 pretreatment level predicts intracranial hemorrhagic complications after thrombolysis in human stroke. Circulation. (2003) 107:598–603. 10.1161/01.CIR.0000046451.38849.90 12566373

[144] Gelderblom M Leypoldt F Steinbach K Behrens D Choe C-U Siler DA Temporal and spatial dynamics of cerebral immune cell accumulation in stroke. Stroke. (2009) 40:1849–57. 10.1161/STROKEAHA.108.534503 19265055

[145] Nadareishvili Z Simpkins AN Hitomi E Reyes D Leigh R. Post-stroke blood-brain barrier disruption and poor functional outcome in patients receiving thrombolytic therapy. Cerebrovasc Dis. (2019) 47:135–42. 10.1159/000499666 30970357 PMC6610790

[146] Ransohoff RM Schafer D Vincent A Blachère NE Bar-Or A. Neuroinflammation: ways in which the immune system affects the brain. Neurotherapeutics. (2015) 12:896–909. 10.1007/s13311-015-0385-3 26306439 PMC4604183

[147] Kamel H Iadecola C. Brain-Immune interactions and ischemic stroke. Arch Neurol. (2012) 69:576–81. 10.1001/archneurol.2011.3590 22782509 PMC3586409

[148] Cheng W Zhao Q Li C Xu Y. Neuroinflammation and brain–peripheral interaction in ischemic stroke: a narrative review. Front Immunol. (2023) 13:1080737. 10.3389/fimmu.2022.1080737 36685518 PMC9849888

[149] Ajmo CT Collier LA Leonardo CC Hall AA Green SM Womble TA Blockade of adrenoreceptors inhibits the splenic response to stroke. Exp Neurol. (2009) 218:47–55. 10.1016/j.expneurol.2009.03.044 19371742 PMC2720830

[150] Pedragosa J Miró-Mur F Otxoa-de-Amezaga A Justicia C Ruíz-Jaén F Ponsaerts P CCR2 deficiency in monocytes impairs angiogenesis and functional recovery after ischemic stroke in mice. J Cereb Blood Flow Metab. (2020) 40:S98–116. 10.1177/0271678X20909055 32151226 PMC7687030

[151] Chen Y Hallenbeck JM Ruetzler C Bol D Thomas K Berman NEJ Overexpression of monocyte chemoattractant protein 1 in the brain exacerbates ischemic brain injury and is associated with recruitment of inflammatory cells. J Cereb Blood Flow Metab. (2003) 23:748–55. 10.1097/01.WCB.0000071885.63724.20 12796723

[152] Michaud J-P Pimentel-Coelho PM Tremblay Y Rivest S. The impact of Ly6Clow monocytes after cerebral hypoxia-ischemia in adult mice. J Cereb Blood Flow Metab. (2014) 34:e1–9. 10.1038/jcbfm.2014.80 24780898 PMC4083393

[153] Auffray C Fogg DK Narni-Mancinelli E Senechal B Trouillet C Saederup N CX3CR1+ CD115+ CD135+ common macrophage/DC precursors and the role of CX3CR1 in their response to inflammation. J Exp Med. (2009) 206:595–606. 10.1084/jem.20081385 19273628 PMC2699130

[154] Chu HX Broughton BRS Ah Kim H Lee S Drummond GR Sobey CG. Evidence that Ly6Chi monocytes are protective in acute ischemic stroke by promoting M2 macrophage polarization. Stroke. (2015) 46:1929–37. 10.1161/STROKEAHA.115.009426 25999385

[155] Cherkashova E Namestnikova D Leonov G Gubskiy I Sukhinich K Melnikov P Comparative study of the efficacy of intra-arterial and intravenous transplantation of human induced pluripotent stem cells-derived neural progenitor cells in experimental stroke. PeerJ. (2023) 11:e16358. 10.7717/peerj.16358 38025691 PMC10640846

[156] Esposito E Ahn BJ Shi J Nakamura Y Park JH Mandeville ET Brain-to-cervical lymph node signaling after stroke. Nat Commun. (2019) 10:5306. 10.1038/s41467-019-13324-w 31757960 PMC6876639

[157] Boisserand LSB Geraldo LH Bouchart J El Kamouh M-R Lee S Sanganahalli BG VEGF-C prophylaxis favors lymphatic drainage and modulates neuroinflammation in a stroke model. J Exp Med. (2024) 221:e20221983. 10.1084/jem.20221983 38442272 PMC10913814

[158] Jiang W Liu X Chen Y Liu M Yuan J Nie M CD4+ CD11b+ T cells infiltrate and aggravate the traumatic brain injury depending on brain-to-cervical lymph node signaling. CNS Neurosci Ther. (2024) 30:e14673. 10.1111/cns.14673 38468459 PMC10928342

[159] Meisel C Meisel A. Suppressing immunosuppression after stroke. N Engl J Med. (2011) 365:2134–6. 10.1056/NEJMcibr1112454 22129259

[160] Simats A Liesz A. Systemic inflammation after stroke: implications for post-stroke comorbidities. EMBO Mol Med. (2022) 14:e16269. 10.15252/emmm.202216269 35971650 PMC9449596

[161] Westendorp WF Nederkoorn PJ Vermeij J-D Dijkgraaf MG van de Beek D. Post-stroke infection: a systematic review and meta-analysis. BMC Neurol. (2011) 11:110. 10.1186/1471-2377-11-110 21933425 PMC3185266

[162] Bergin SP Coles A Calvert SB Farley J Powers JH Zervos MJ PROPHETIC: prospective identification of pneumonia in hospitalized patients in the ICU. Chest. (2020) 158:2370–80. 10.1016/j.chest.2020.06.034 32615191 PMC7722207

[163] Kalra L Irshad S Hodsoll J Simpson M Gulliford M Smithard D Prophylactic antibiotics after acute stroke for reducing pneumonia in patients with dysphagia (STROKE-INF): a prospective, cluster-randomised, open-label, masked endpoint, controlled clinical trial. Lancet. (2015) 386:1835–44. 10.1016/S0140-6736(15)00126-9 26343840

[164] Westendorp WF Vermeij J-D Zock E Hooijenga IJ Kruyt ND Bosboom HJLW The preventive antibiotics in stroke study (PASS): a pragmatic randomised open-label masked endpoint clinical trial. Lancet. (2015) 385:1519–26. 10.1016/S0140-6736(14)62456-9 25612858

[165] Römer C Engel O Winek K Hochmeister S Zhang T Royl G Blocking stroke-induced immunodeficiency increases CNS antigen-specific autoreactivity but does not worsen functional outcome after experimental stroke. J Neurosci. (2015) 35:7777–94. 10.1523/JNEUROSCI.1532-14.2015 25995466 PMC6795191

[166] Haeusler KG Schmidt WUH Föhring F Meisel C Helms T Jungehulsing GJ Cellular immunodepression preceding infectious complications after acute ischemic stroke in humans. Cerebrovasc Dis. (2008) 25:50–8. 10.1159/000111499 18033958

[167] Urra X Cervera A Obach V Climent N Planas AM Chamorro A. Monocytes are major players in the prognosis and risk of infection after acute stroke. Stroke. (2009) 40:1262–8. 10.1161/STROKEAHA.108.532085 19164783

[168] Bernardo-Castro S Sousa JA Brás A Cecília C Rodrigues B Almendra L Pathophysiology of blood-brain barrier permeability throughout the different stages of ischemic stroke and its implication on hemorrhagic transformation and recovery. Front Neurol. (2020) 11:594672. 10.3389/fneur.2020.594672 33362697 PMC7756029

[169] Liebner S Dijkhuizen RM Reiss Y Plate KH Agalliu D Constantin G. Functional morphology of the blood-brain barrier in health and disease. Acta Neuropathol. (2018) 135:311–36. 10.1007/s00401-018-1815-1 29411111 PMC6781630

[170] Lin Z Li Y Su P Mao D Wei Z Pillai JJ Non-contrast MR imaging of blood-brain-barrier permeability to water. Magn Reson Med. (2018) 80:1507–20. 10.1002/mrm.27141 29498097 PMC6097906

[171] Moyaert P Padrela BE Morgan CA Petr J Versijpt J Barkhof F Imaging blood-brain barrier dysfunction: a state-of-the-art review from a clinical perspective. Front Aging Neurosci. (2023) 15:1132077. 10.3389/fnagi.2023.1132077 37139088 PMC10150073

[172] Bani-Sadr A Mechtouff L De Bourguignon C Mauffrey A Boutelier T Cho T-H Blood-brain barrier permeability and kinetics of inflammatory markers in acute stroke patients treated with thrombectomy. Neurology. (2023) 101:e502–11. 10.1212/WNL.0000000000207460 37290975 PMC10401692

[173] Bivard A Kleinig T Churilov L Levi C Lin L Cheng X Permeability measures predict hemorrhagic transformation after ischemic stroke. Ann Neurol. (2020) 88:466–76. 10.1002/ana.25785 32418242 PMC7496077

[174] Protzmann J Jung F Jakobsson L Fredriksson L. Analysis of ischemic stroke-mediated effects on blood–brain barrier properties along the arteriovenous axis assessed by intravital two-photon imaging. Fluids Barriers CNS. (2024) 21:35. 10.1186/s12987-024-00537-5 38622710 PMC11017501

[175] Dickens AM Vainio S Marjamäki P Johansson J Lehtiniemi P Rokka J Detection of microglial activation in an acute model of neuroinflammation using PET and radiotracers 11C-(R)-PK11195 and 18F-GE-180. J Nucl Med. (2014) 55:466–72. 10.2967/jnumed.113.125625 24516258

[176] Cumming P Burgher B Patkar O Breakspear M Vasdev N Thomas P Sifting through the surfeit of neuroinflammation tracers. J Cereb Blood Flow Metab. (2018) 38:204–24. 10.1177/0271678X17748786 29256293 PMC5951023

[177] Stoll G Bendszus M. New approaches to neuroimaging of central nervous system inflammation. Curr Opin Neurol. (2010) 23:282–6. 10.1097/WCO.0b013e328337f4b5 20168228

[178] Deddens LH Van Tilborg GAF Mulder WJM De Vries HE Dijkhuizen RM. Imaging neuroinflammation after stroke: current status of cellular and molecular MRI strategies. Cerebrovasc Dis. (2012) 33:392–402. 10.1159/000336116 22456323

[179] Tuwar MN Chen W-H Chiwaya AM Yeh H-L Nguyen MH Bai C-H. Brain-derived neurotrophic factor (BDNF) and translocator protein (TSPO) as diagnostic biomarkers for acute ischemic stroke. Diagnostics. (2023) 13:2298. 10.3390/diagnostics13132298 37443691 PMC10340661

[180] Van Camp N Lavisse S Roost P Gubinelli F Hillmer A Boutin H. TSPO imaging in animal models of brain diseases. Eur J Nucl Med Mol Imaging. (2021) 49:77–109. 10.1007/s00259-021-05379-z 34245328 PMC8712305

[181] Helgebostad R Revheim M-E Johnsrud K Amlie K Alavi A Connelly JP. Clinical applications of somatostatin receptor (agonist) PET tracers beyond neuroendocrine tumors. Diagnostics. (2022) 12:528. 10.3390/diagnostics12020528 35204618 PMC8870812

[182] Baba O Huang L-H Elvington A Szpakowska M Sultan D Heo GS CXCR4-binding PET tracers link monocyte recruitment and endothelial injury in murine atherosclerosis. Arterioscler Thromb Vasc Biol. (2021) 41:822–36. 10.1161/ATVBAHA.120.315053 33327748 PMC8105279

[183] Thayse K Kindt N Laurent S Carlier S. VCAM-1 target in non-invasive imaging for the detection of atherosclerotic plaques. Biology. (2020) 9:368. 10.3390/biology9110368 33138124 PMC7692297

[184] Gauberti M Fournier AP Docagne F Vivien D Martinez de Lizarrondo S. Molecular magnetic resonance imaging of endothelial activation in the central nervous system. Theranostics. (2018) 8:1195–212. 10.7150/thno.22662 29507614 PMC5835930

[185] Banerjee A McCullough LD. Sex-specific immune responses in stroke. Stroke. (2022) 53:1449–59. 10.1161/STROKEAHA.122.036945 35468002 PMC9668253

[186] Liu J Sato Y Falcone-Juengert J Kurisu K Shi J Yenari MA. Sexual dimorphism in immune cell responses following stroke. Neurobiol Dis. (2022) 172:105836. 10.1016/j.nbd.2022.105836 35932990

[187] Tariq MB Lee J McCullough LD. Sex differences in the inflammatory response to stroke. Semin Immunopathol. (2023) 45:295–313. 10.1007/s00281-022-00969-x 36355204 PMC10924671

[188] Ahnstedt H McCullough LD. The impact of sex and age on T cell immunity and ischemic stroke outcomes. Cell Immunol. (2019) 345:103960. 10.1016/j.cellimm.2019.103960 31519365 PMC6832888

[189] Villapol S Loane DJ Burns MP. Sexual dimorphism in the inflammatory response to traumatic brain injury. Glia. (2017) 65:1423–38. 10.1002/glia.23171 28608978 PMC5609840

[190] McCullough LD Mirza MA Xu Y Bentivegna K Steffens EB Ritzel R Stroke sensitivity in the aged: sex chromosome complement vs. gonadal hormones. Aging. (2016) 8:1432–41. 10.18632/aging.100997 27405096 PMC4993340

[191] Mirza MA Ritzel R Xu Y McCullough LD Liu F. Sexually dimorphic outcomes and inflammatory responses in hypoxic-ischemic encephalopathy. J Neuroinflammation. (2015) 12:32. 10.1186/s12974-015-0251-6 25889641 PMC4359482

[192] Ahnstedt H Patrizz A Chauhan A Roy-O’Reilly M Furr JW Spychala MS Sex differences in T cell immune responses, gut permeability and outcome after ischemic stroke in aged mice. Brain Behav Immun. (2020) 87:556–67. 10.1016/j.bbi.2020.02.001 32058038 PMC7590503

[193] Perl M Chung C-S Perl U Biffl WL Cioffi WG Ayala A. Beneficial versus detrimental effects of neutrophils are determined by the nature of the insult. J Am Coll Surg. (2007) 204:840–52; discussion 852–853. 10.1016/j.jamcollsurg.2007.02.023 17481496

[194] Meier A Chang JJ Chan ES Pollard RB Sidhu HK Kulkarni S Sex differences in the toll-like receptor-mediated response of plasmacytoid dendritic cells to HIV-1. Nat Med. (2009) 15:955–9. 10.1038/nm.2004 19597505 PMC2821111

[195] Guneykaya D Ivanov A Hernandez DP Haage V Wojtas B Meyer N Transcriptional and translational differences of microglia from male and female brains. Cell Rep. (2018) 24:2773–83.e6. 10.1016/j.celrep.2018.08.001 30184509

[196] Ugidos IF Pistono C Korhonen P Gómez-Budia M Sitnikova V Klecki P Sex differences in poststroke inflammation: a focus on microglia across the lifespan. Stroke. (2022) 53:1500–9. 10.1161/STROKEAHA.122.039138 35468000

[197] Thion MS Low D Silvin A Chen J Grisel P Schulte-Schrepping J Microbiome influences prenatal and adult microglia in a sex-specific manner. Cell. (2018) 172:500–16.e16. 10.1016/j.cell.2017.11.042 29275859 PMC5786503

[198] Xu H Ge Y Liu Y Zheng Y Hu R Ren C Identification of the key genes and immune infiltrating cells determined by sex differences in ischaemic stroke through co-expression network module. IET Syst Biol. (2022) 16:28–41. 10.1049/syb2.12039 34792838 PMC8849259

[199] Lasek-Bal A Jedrzejowska-Szypulka H Student S Warsz-Wianecka A Zareba K Puz P The importance of selected markers of inflammation and blood-brain barrier damage for short-term ischemic stroke prognosis. J Physiol Pharmacol. (2019) 70:209–17. 10.26402/jpp.2019.2.04 31356182

[200] Dutta D Dutta D. Crossroads between cognitive connectomics and sociomics: synergies and squabbles amidst two omics. Available online at: https://services.igi-global.com/resolvedoi/resolve.aspx?doi=104018/979-8-3693-1265-0.ch017 (Accessed August 08, 2024).

[201] Cao Y Yue X Jia M Wang J. Neuroinflammation and anti-inflammatory therapy for ischemic stroke. Heliyon. (2023) 9:e17986. 10.1016/j.heliyon.2023.e17986 37519706 PMC10372247

[202] Mattila OS Ashton NJ Blennow K Zetterberg H Harve-Rytsälä H Pihlasviita S Ultra-early differential diagnosis of acute cerebral ischemia and hemorrhagic stroke by measuring the prehospital release rate of GFAP. Clin Chem. (2021) 67:1361–72. 10.1093/clinchem/hvab128 34383905

[203] Lakhan SE Kirchgessner A Tepper D Leonard A. Matrix metalloproteinases and blood-brain barrier disruption in acute ischemic stroke. Front Neurol. (2013) 4:32. 10.3389/fneur.2013.00032 23565108 PMC3615191

[204] Turner RJ Sharp FR. Implications of MMP9 for blood brain barrier disruption and hemorrhagic transformation following ischemic stroke. Front Cell Neurosci. (2016) 10:56. 10.3389/fncel.2016.00056 26973468 PMC4777722

[205] Ahn JW Hwang J Lee M Kim JH Cho H-J Lee H-W Serum neurofilament light chain levels are correlated with the infarct volume in patients with acute ischemic stroke. Medicine. (2022) 101:e30849. 10.1097/MD.0000000000030849 36181119 PMC9524991

[206] Eyileten C Wicik Z De Rosa S Mirowska-Guzel D Soplinska A Indolfi C MicroRNAs as diagnostic and prognostic biomarkers in ischemic stroke—a comprehensive review and bioinformatic analysis. Cells. (2018) 7:249. 10.3390/cells7120249 30563269 PMC6316722

[207] Jickling GC Sharp FR. Biomarker panels in ischemic stroke. Stroke. (2015) 46:915–20. 10.1161/STROKEAHA.114.005604 25657186 PMC4342265

[208] Baez SC García Del Barco D Hardy-Sosa A Guillen Nieto G Bringas-Vega ML Llibre-Guerra JJ Scalable bio marker combinations for early stroke diagnosis: a systematic review. Front Neurol. (2021) 12:638693. 10.3389/fneur.2021.638693 34122297 PMC8193128

[209] Sharp FR Jickling GC. Modelling immunity and inflammation in stroke: differences between rodents and humans? Stroke. (2014) 45:e179–80. 10.1161/STROKEAHA.114.005639 25061082 PMC4215953

[210] Seok J Warren HS Cuenca AG Mindrinos MN Baker HV Xu W Genomic responses in mouse models poorly mimic human inflammatory diseases. Proc Natl Acad Sci U S A. (2013) 110:3507–12. 10.1073/pnas.1222878110 23401516 PMC3587220

